# Harmful Freshwater Algal Blooms, With an Emphasis on Cyanobacteria

**DOI:** 10.1100/tsw.2001.16

**Published:** 2001-04-04

**Authors:** Hans W. Paerl, Rolland S. Fulton, Pia H. Moisander, Julianne Dyble

**Affiliations:** Institute of Marine Sciences, University of North Carolina at Chapel Hill, Morehead City, NC 28557, USA

## Abstract

Suspended algae, or phytoplankton, are the prime source of organic matter supporting food webs in freshwater ecosystems. Phytoplankton productivity is reliant on adequate nutrient supplies; however, increasing rates of nutrient supply, much of it manmade, fuels accelerating primary production or eutrophication. An obvious and problematic symptom of eutrophication is rapid growth and accumulations of phytoplankton, leading to discoloration of affected waters. These events are termed blooms. Blooms are a prime agent of water quality deterioration, including foul odors and tastes, deoxygenation of bottom waters (hypoxia and anoxia), toxicity, fish kills, and food web alterations. Toxins produced by blooms can adversely affect animal (including human) health in waters used for recreational and drinking purposes. Numerous freshwater genera within the diverse phyla comprising the phytoplankton are capable of forming blooms; however, the blue-green algae (or cyanobacteria) are the most notorious bloom formers. This is especially true for harmful toxic, surface-dwelling, scum-forming genera (e.g., Anabaena, Aphanizomenon, Nodularia, Microcystis) and some subsurface bloom-formers (Cylindrospermopsis, Oscillatoria) that are adept at exploiting nutrient-enriched conditions. They thrive in highly productive waters by being able to rapidly migrate between radiance-rich surface waters and nutrient-rich bottom waters. Furthermore, many harmful species are tolerant of extreme environmental conditions, including very high light levels, high temperatures, various degrees of desiccation, and periodic nutrient deprivation. Some of the most noxious cyanobacterial bloom genera (e.g., Anabaena, Aphanizomenon, Cylindrospermopsis, Nodularia) are capable of fixing atmospheric nitrogen (N_2_), enabling them to periodically dominate under nitrogen-limited conditions. Cyanobacteria produce a range of organic compounds, including those that are toxic to higher-ranked consumers, from zooplankton to further up the food chain. Both N_2_- and non-N_2_-fixing genera participate in mutualistic and symbiotic associations with microorganisms, higher plants, and animals. These associations appear to be of great benefit to their survival and periodic dominance. In this review, we address the ecological impacts and environmental controls of harmful blooms, with an emphasis on the ecology, physiology, and management of cyanobacterial bloom taxa. Combinations of physical, chemical, and biotic features of natural waters function in a synergistic fashion to determine the sensitivity of water bodies. In waters susceptible to blooms, human activities in water- and airsheds have been linked to the extent and magnitudes of blooms. Control and management of cyanobacterial and other phytoplankton blooms invariably includes nutrient input constraints, most often focused on nitrogen (N) and/or phosphorus (P). The types and amount of nutrient input constraints depend on hydrologic, climatic, geographic, and geologic factors, which interact with anthropogenic and natural nutrient input regimes. While single nutrient input constraints may be effective in some water bodies, dual N and P input reductions are usually required for effective long-term control and management of harmful blooms. In some systems where hydrologic manipulations (i.e., plentiful water supplies) are possible, reducing the water residence time by enhanced flushing and artificial mixing (in conjunction with nutrient input constraints) can be particularly effective alternatives. Implications of various management strategies, based on combined ecophysiological and environmental considerations, are discussed.

### TABLE OF CONTENTS

**Introduction** ........ 77
Why do Blooms Occur? ........ 77
What Makes a Bloom Harmful? ........ 77
Who are the Players? ........ 78
Chlorophyta (Green Algae) ........ 78
Dinophyta (Dinoflagellates) ........ 78
Cryptophyta ........ 80
Chrysophyta ........ 83
Cyanobacteria ........ 83

**Harmful Cyanobacterial Bloom Dynamics** ........ 84
Nitrogen and Phosphorus ........ 85
Organic Matter ........ 90
Iron and Trace Elements ........ 91
Conductivity and Salinity ........ 92
Turbulence ........ 93
Genetic Constraints on Cyanobacterial Expansion ........ 94
Biotic Interactions ........ 95
Interactions with Other Microbes ........ 95
Interactions with Competitors and Consumers ........ 101
Effects on Fish and Higher Trophic Levels ........ 102
Domestic Animal and Human Health Effects of Cyanotoxins ........ 103

**Harmful Bloom Management** ........ 103
Phosphorus Management ........ 104
Nitrogen Management ........ 105

**Concluding Remarks** ........ 106

**Acknowledgements** ........ 106

**References** ........ 106

## INTRODUCTION

### Why do Blooms Occur?

Microscopic suspended algae, or phytoplankton, are of fundamental importance in supporting the productivity of aquatic ecosystems. Phytoplankton utilize light, carbon dioxide, and a range of inorganic and organic nutrients to photosynthetically produce biomass, which provides the energy and materials at the base of food webs. In most water bodies, there is a fine balance between adequate irradiance and nutrient supply that determines the rate of production of phytoplankton biomass, or primary productivity[1,2,3]. The regulation of phytoplankton production by light and nutrient availability is often referred to as "bottom-up" control. Newly produced phytoplankton biomass is consumed by planktonic herbivores, which by grazing or predation exert a "top-down" control on phytoplankton production. The balance between bottom-up and top-down control determines how much *net* production takes place. Physical factors such as vertical mixing and the rate of flushing (i.e., residence time) in a water body exert additional controls on how much phytoplankton production and consumption can take place over time and space[4,5,6]. The interaction between physical, chemical, and biotic factors determines environmental heterogeneity, a key factor determining the diversity and successional patterns of phytoplankton[2,7,8]. Under optimal growth conditions, high rates of primary productivity take place. If consumption cannot keep pace with production, excess phytoplankton accumulates. Phytoplankton accumulations that lead to visible discoloration (most often yellow, green, blue-green, red, or brown) of the water are commonly referred to as blooms[9,10,11]. Phytoplankton blooms can occur at the surface or at specific depths in the water column corresponding to optimal light and nutrient levels as well as thermal and density stratification. Blooms can accumulate passively through buoyancy compensation, or they may accumulate through active mechanisms, including high growth rates and swimming migration. From an ecosystem perspective, high rates of nutrient loading accompanied by water residence time long enough to support adequate growth and high reproductive rates, combined with relatively low grazing rates, represent optimal conditions for phytoplankton bloom development[2,4,9,12,13].

### What Makes a Bloom Harmful?

Blooms accumulate, proliferate, and manifest themselves in myriad ways. Many blooms accumulate near the water's surface, where light is plentiful[9,10]. Rapid bloom development can result if growth and division rates are fast or, in the case of buoyant species, when stable water column conditions (i.e., lack of mixing) facilitate surface accumulations. Accumulations may lead to adverse tastes and odors as well as toxicity of affected waters[9,14]. At high cell densities, blooms may rapidly deplete nutrients, increase turbidity (and hence decrease transparency), exhaust inorganic carbon ($CO_2$) supplies and other essential resources, thus causing a sudden decline in biomass, referred to as a "crash"[9,11]. This usually involves decaying, odoriferous, unsightly scums. Scums can harbor a variety of microbial pathogens and rob the underlying waters of oxygen, causing significant chemical and biological changes, including hypoxia (dissolved oxygen concentrations less than 4 mg $l^{-1}$, stressful to most fauna), anoxia (no detectable oxygen, fatal to most finfish and shellfish), release of toxic hydrogen sulfide, and accelerated release of nutrients from sediments which further aggravates eutrophication and blooms[8,10,13]. Waters impacted in this matter are often off-limits to recreational use (bathing, fishing). In addition, some bloom-forming species produce compounds toxic to biota including invertebrates (e.g., zooplankton), finfish and shellfish, and vertebrate consumers of drinking water, including humans[14,15]. These conditions are undesirable and noxious from water use, recreational and health perspectives; hence the designation "harmful".

### Who are the Players?

Most major freshwater algal phyla contain some harmful or otherwise nuisance bloom genera (Table 1, Figs. 1 and 2). These include the eukaryotic green algae or chlorophytes, dinoflagellates, cryptophytes, chrysophytes (including diatoms), and the prokaryotic blue-green algae or cyano-bacteria[2,16,17]. These groups include taste and odor producers, toxin producers, food web disrupters, and hypoxia-generating and aesthetically undesirable types (freshwater "red tides", scum-formers). Examples are discussed below. Cyano-bacteria, by far the most notorious and problematic freshwater group, is the detailed subject of this review.

#### *Chlorophyta (Green Algae)*

Members of the phylum Chlorophyta, or green algae, are inhabitants of geographically diverse lakes, reservoirs, streams, and rivers varying in trophic state. Chlorophytes include microscopic planktonic (solitary, colonial, or filamentous forms), attached filamentous, and macroalgal genera. While some macroalgal blooms can cause water quality problems in the littoral zones of lakes, rivers, and estuaries, chlorophytes are normally not thought of as nuisance algae. However, nutrient-enriched conditions favor members of this group, and high chlorophyte density is indicative of eutrophying conditions. In highly eutrophic waters, green algae often codominate with bloom-forming cyanobacteria (see below). However, in contrast to the cyanobacteria, there is no documentation of toxicity among chlorophytes.

Some chlorophyte genera form distinctive blooms that on rare occasions may proliferate and persist to cause water quality problems such as hypoxia, tastes, and odors[2]. Members of the genus *Botryococcus* can form highly buoyant surface-dwelling aggregates that can accumulate as scums[18]. While these surface blooms are aesthetically detractive, they are not necessarily indicative of excessive nutrient loading or water quality deterioration. For example, *Botryococcus* blooms regularly occur in the surface waters of Lake Taupo, New Zealand's largest lake, located in the middle of the North Island (Paerl, unpublished). This oligotrophic lake has excellent water quality and a world-class trout fishery. Other genera that can grow to high enough abundance to cause discoloration of inland waters include *Sphaerocystis, Dictyosphaerium*, *Scenedesmus,* and *Chlorococcus*.

#### *Dinophyta (Dinoflagellates)*

This phylum is composed of highly motile single-celled taxa, some of which are armored and others not. Dinoflagellates are almost exclusively planktonic, preferring well-illuminated near-surface waters[2,19]. Most dinoflagellates are pigmented (chlorophyll and carotenoids) and grow photosynthetically; however, there are nonpigmented genera that must support growth heterotrophically, either by directly utilizing dissolved organic matter or by ingesting small food particles, including bacteria and other algae[20]. Dinoflagellates are widely distributed in freshwater environments. They may be found over a broad range of trophic states, spanning oligotrophy to hypereutrophy.

Accelerated growth and dense accumulations of dinoflagellates in near-surface waters can cause blooms. As with other bloom-forming phytoplankton, nutrient-enriched conditions tend to enhance growth and bloom potential. However, there is substantial variability in the extent to which blooms are related to trophic state of affected waters. In some large lakes, blooms can occur even under low nutrient-loading conditions. A key to the success of bloom-forming dinoflagellates is their ability to rapidly migrate between nutrient-rich deeper waters and radiance-rich surface waters, thereby satisfying both nutrient and light requirements[6,19].

Dinoflagellate blooms can manifest themselves at high densities (>100,000 cells/ml) and in many colors, including pale green, yellow, red, and brown. The armored genera *Ceratium*, *Heterocapsa*, *Gymnodinium*, and *Peridinium* can, by

**TABLE 1**
**Representative Freshwater Nuisance A**

| Kingdom/Phylum | Genus | Preferred Bloom Conditions |
|---|---|---|
| Prokaryota | | |
| Cyanobacteria | | |
| ($N_2$-fixing) | *Anabaena* | P-enriched, warm, stratified, long-residence |
| | *Aphanizomenon* | time, high irradiance, eutrophic |
| | *Cylindrospermopsis* | |
| | *Gloeotrichia* | |
| | *Nodularia* | |
| (Non-$N_2$-fixing) | *Microcystis* | N- and P-enriched, eutrophic conditions, |
| | *Oscillatoria* | warm, stratified, long residence time |
| | *Gomphosphaeria* | |
| Eukaryota | | |
| Chlorophyta | *Botryococcus* | Moderate N- and P-enriched, stratified, high irradiance |
| | *Chlorococcus* | Eutrophic, N- and P-enriched |
| | *Sphaerocystis* | |
| Pyrrhophyta (Dinophyta) | *Ceratium* | N- and P-enriched, stratified, some |
| | *Peridinium* | oligohaline |
| | *Heterocapsa* | |
| | *Prorocentrum* | |
| Cryptophyta | *Cryptomonas* | N- and P-enriched, eutrophied, |
| | *Rhodomonas* | fresh to oligohaline, stratified |
| Chrysophyta | *Chromulina* | N- and P-enriched, toxic at high N |
| | *Chrysochromulina* | enrichment, stratified |
| | *Dinobryon* | |
| | *Mallomonas* | |
| | *Prymnesium* | |

virtue of high densities and rich carotenoid content, form spectacular blooms often referred to as freshwater "red tides". These blooms can, at times, lead to massive accumulations of decaying biomass in surface waters, and when blown ashore or trapped in shallow embayments, can cause water quality problems including foul odors and tastes, hypoxia, and loss of aesthetic and recreational value of affected waters. While marine analog genera (including *Alexandrium, Gymnodinium*, *Heterosigma*, and *Noctiluca*) are well-known toxin producers negatively affecting a wide array of biota ranging from invertebrates to fish to large mammals and humans, freshwater dinoflagellates are generally nontoxic. Some toxic and hypoxia-generating nuisance dinoflagellate species flourish under brackish estuarine conditions (*Dinophysis*), where blooms can cause substantial ecological and economic (fisheries) losses. Included are periodic outbreaks of *Prorocentrum* in the Chesapeake Bay, and *Gymnodium, Gyrodinium,* and *Heterocapsa* in North Carolina estuaries. These blooms have, at times, been linked to excessive inputs of both nitrogen (N) and phosphorus (P).

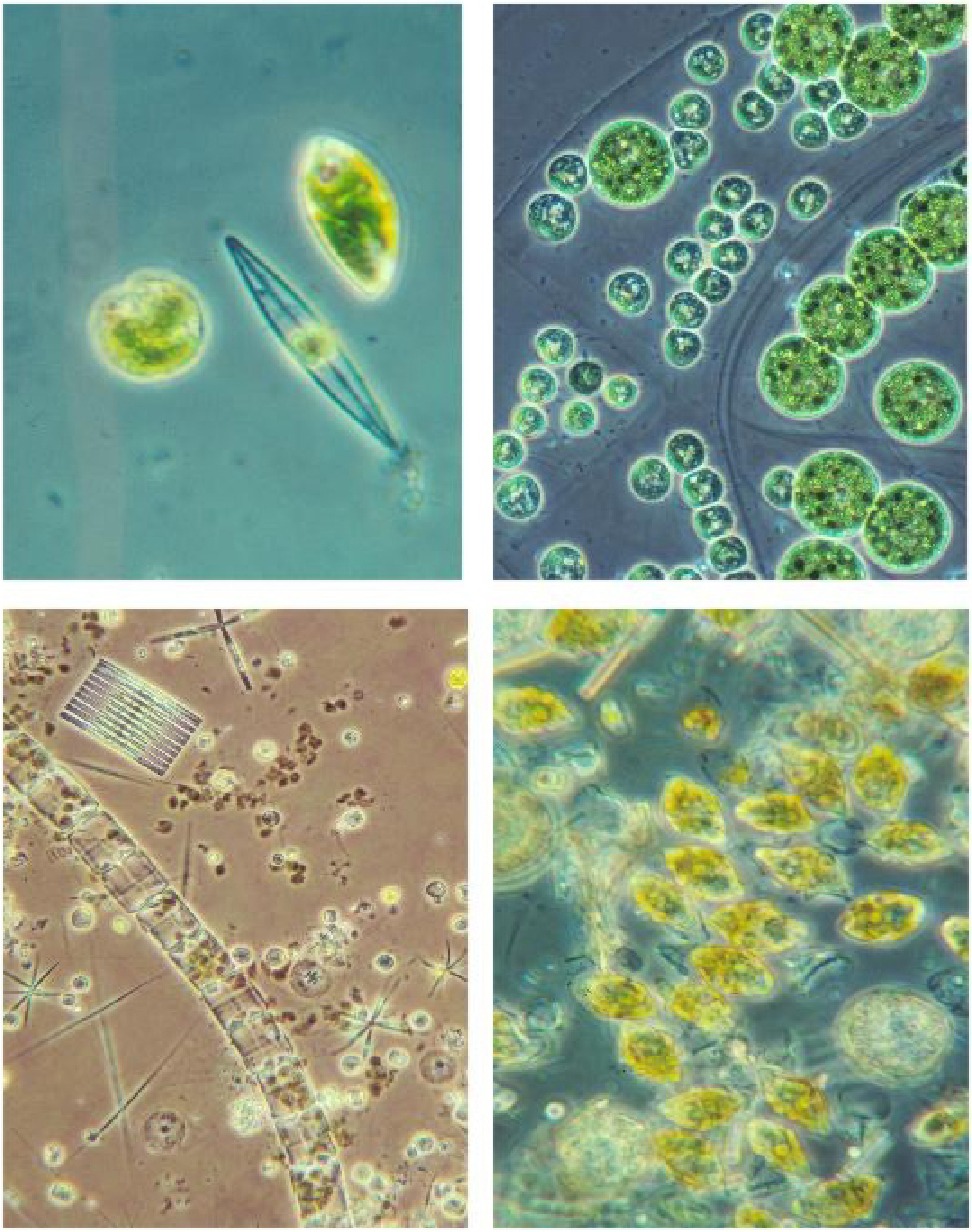

**FIGURE 1**. Representative photomicrographs of eukaryotic algal phyla containing bloom-forming species. These examples are from natural populations in the Neuse River Estuary, North Carolina. Upper left, cryptomonads and a diatom; upper right, chlorophytes or green algae; lower left, chain-forming diatoms; lower right, dinoflagellates.

### Cryptophyta

Cryptophytes are motile planktonic algae that grow phototrophically[2]. This group is broadly distributed in lakes, slow-moving rivers, and oligohaline regions of estuaries, but less so in fast-moving streams[21]. Cryptophytes generally prefer nutrient-enriched waters ranging from mesotrophic to hypereutrophic. High densities of the genera

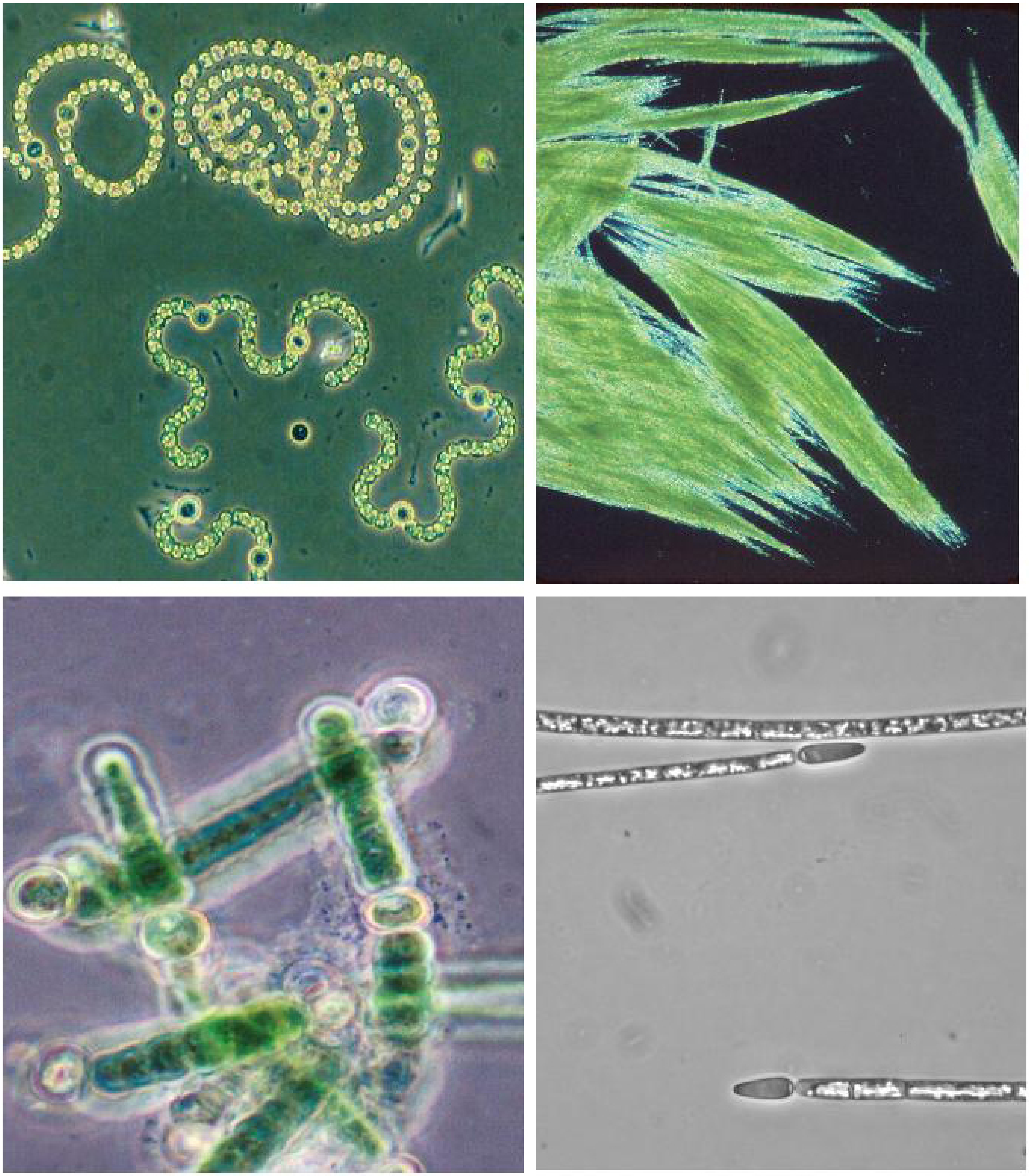

**(2A)**

**FIGURE 2**. Major cyanobacterial bloom-forming genera. (A) Members of heterocystous, $N_2$-fixing genera. Upper left, *Anabaena flos-aquae* (top of frame) and *A. spiroides*; upper right, flake-like colonies of *Aphanizomenon flos-aquae*; lower left, *Nodularia* sp.; lower right, *Cylindrospermopsis raciborskii*. (B) Members of nonheterocystous genera. Upper left, *Microcystis aeruginosa* colony; upper right, unidentified colonial coccoid cyanobacteria; lower left, *Oscillatoria* sp. filaments; lower right, aggregated *Lyngbya* sp. filaments.

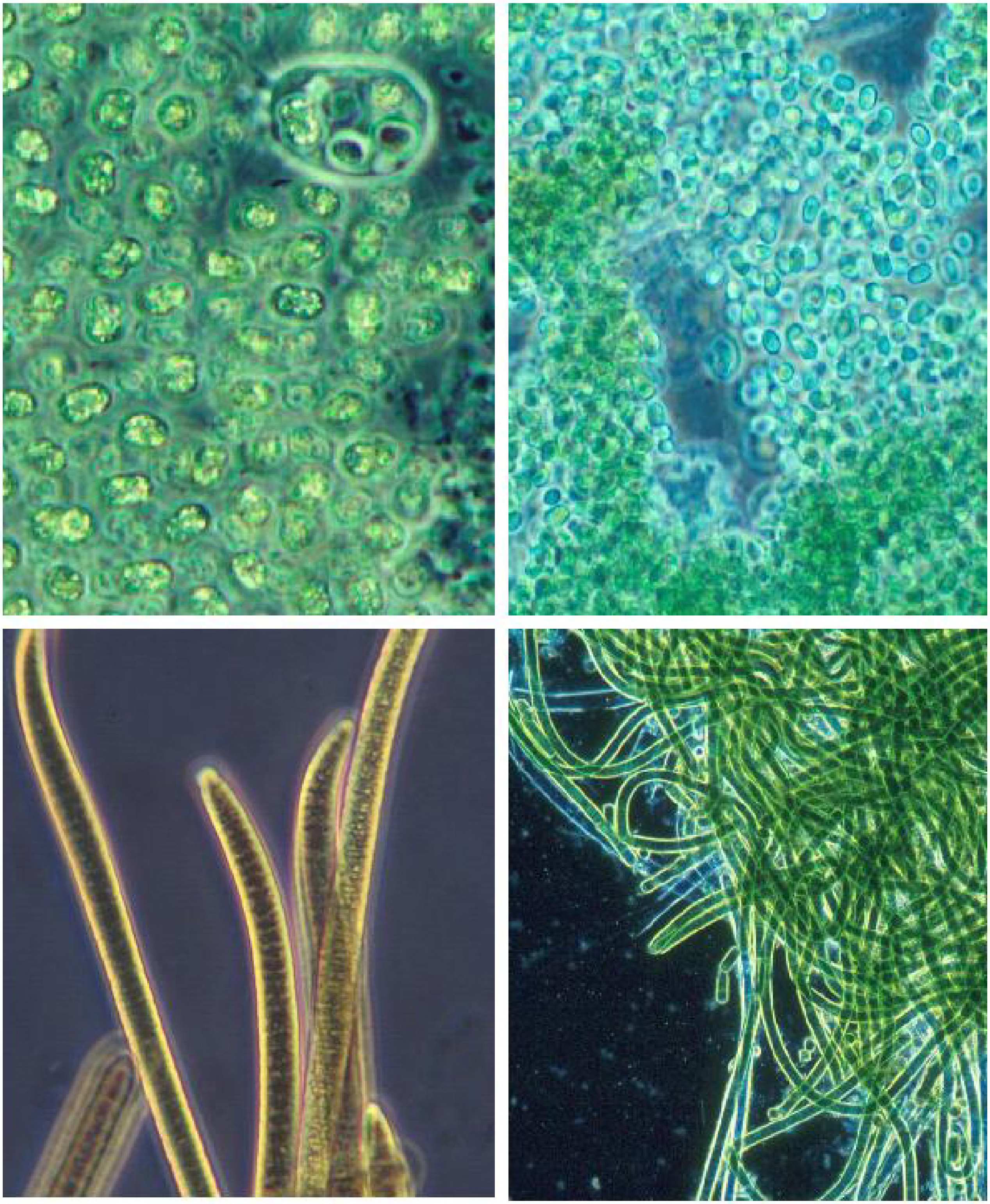

(FIGURE 2B)

*Rhodomonas* and *Cryptomonas* can occur following periods of nutrient enrichment. The discoloration resulting from *Rhodomonas* and *Cryptomonas* blooms can be traced to abundant cellular carotenoid pigments. In some systems, these blooms are indicative of enhanced nutrient loading. Typically, *Cryptomonas* blooms occur from late winter through spring, giving way to diatom, chlorophyte, dinoflagellate, and cyanobacterial dominance in summer and fall. When combined with dinoflagellates, cryptomonad blooms can be responsible for freshwater "red tides", localized hypoxia, and adverse aesthetics. As a group, cryptophytes are not known to be toxic to either resident biota or humans.

### Chrysophyta

The chrysophytes consist of phototrophic, unicellular, colonial, filamentous, or siphonaceous genera, all of which contain abundant colored (usually golden to brown) carotenoid pigments. All genera are planktonic, many are motile, propelled by either flagella (classes: Chrysophyceae, Haptophyceae, and Craspedophyceaea) (*Chromulina, Chrysochromulina, Prymnesium*) or mucilage excretions ("jet propulsion"), as is the case among the diatoms (class Bacillariophyceae)[2]. Others are nonmotile, either solitary or colonial (*Dinobryon, Mallomonas, Synura*). Chrysophytes exhibit broad growth requirements and can be found in diverse freshwater habitats.

Few nuisance taxa exist; however, among these, water quality problems have been reported. Dense blooms of mucilage-producing diatoms can foul shorelines, and large accumulations of diatom biomass can exacerbate hypoxia. Such blooms can be indicative of enhanced nutrient loading, although they are not nearly as diagnostic as cyanobacterial and green algal blooms. In both fresh and brackish nutrient-enriched waters, *Prymnesium* can accumulate in large numbers. Members of this genus produce a potent toxin that affects gill-bearing animals, including fish and mollusks. This genus has posed serious water quality problems in aquaculture ponds, especially those in tropical locations where the combined effects of high temperature, stagnancy, and toxic *Prymnesium* can lead to severe fish kills. In the brackish waters of the western Baltic Sea, the Danish sounds, and Kattegat-Skagerrak (near the entrance to the North Sea) large and persistent blooms of *Chrysochromulina polylepis* have been reported in response to periods of elevated nutrient (especially nitrogen) loading[22]. These blooms have proven toxic to fish and shellfish; an unusually large bloom in 1988 was shown to be responsible for devastating the salmon net pen aquaculture industry in the Kattegat region between southwestern Sweden and southern Norway[22]. Toxicity in *C. polylepis* appears to be strongly stimulated by nitrogen enrichment relative to phosphorus, leading to increased phosphorus limitation. These problems can occur in brackish and full-salinity aquaculture ponds[23], but have not been reported in freshwater environments.

### Cyanobacteria

The cyanobacteria (blue-green algae) are the most widespread and problematic freshwater nuisance algal taxa. Cyanobacteria are prokaryotic, i.e., they have no defined nucleus and organelles; their cellular structure is most similar to bacteria. In freshwater environments, cyanobacteria can be found in three basic morphological groups: 1) unicells, which may be solitary or aggregated in colonies; 2) undifferentiated, nonheterocystous filaments, which also may be solitary or aggregated; and 3) filamentous forms containing differentiated cells called heterocysts (Fig. 2). Each of these groups contains nuisance taxa (Table 2, Fig. 2) that will be discussed in detail below. Cyanobacteria are the oldest (~2.5 billion years) oxygenic phototrophic inhabitants on Earth. A long evolutionary history has endowed them with an array of physiological, morphological, and ecological adaptations in response to geochemical and climatic change[24,25,26]. Cyanobacteria are also uniquely adapted to nutrient deficiencies and alterations. For example, they are the only phytoplankton group capable of utilizing atmospheric dinitrogen gas ($N_2$) as a nitrogen source via biological $N_2$ fixation ($N_2 + 6H^+$® $2NH_3$) and as such can circumvent N-limited conditions. In addition, they are capable of taking up phosphorus (P) in excess of cellular growth requirements (luxury consumption), and storing it for subsequent use under P-limited conditions. These physiological traits allow cyanobacteria to exploit both nutrient-deficient and -enriched environments[9,10,13,27].

The most visible and troubling aspect of cyanobacterial opportunism is the development and proliferation of surface and subsurface nuisance blooms in nutrient-enriched freshwater and brackish ecosystems[9,13,28] (Fig. 3). Because they may be toxic, blooms can pose serious water quality, fisheries resource, aquaculture, and animal and human health problems[29,30,31]. Blooms have been linked to liver disease, human cancers, and deaths[31,32] (Table 3). In addition, blooms can cause hypoxia and anoxia of underlying waters, which may lead to fish and bottom fauna mortalities[27,33]. Recreational use and aesthetic values of affected waters may be seriously impaired. In addition, nutrient (C, N, P, iron, and trace elements) fluxes and biogeochemical cycling can be impacted by high rates of $CO_2$ and $N_2$ fixation and massive amounts of biomass generated and sedimented during blooms[34,35,36].

**TABLE 2**
**Name and Producer Organism for the Cyanobacterial Toxins**

| Name | Produced by |
|---|---|
| Biotoxins | |
| Anatoxin-a | *Anabaena, Aphanizomenon, Oscillatoria (Planktothrix)* |
| Homo-Anatoxin-a | |
| Anatoxin-a(s) | *Anabaena, Oscillatoria (Planktothrix)* |
| Cylindrospermopsin | *Aphanizomenon, Cylindrospermopsis, Umezakia* |
| Microcystins | *Anabaena, Aphanocapsa, Hapalosiphon, Microcystis, Nostoc, Oscillatoria (Planktothrix)* |
| Nodularins | *Nodularia* (brackish water) |
| Paralytic Shellfish Poisons (Saxitoxins) | *Anabaena, Aphanizomenon, Cylindrospermopsis, Lyngbya* |
| Debromoaplysiatoxin, | |
| Lyngbyatoxin | *Lyngbya* (marine) |
| Aplysiatoxin | *Schizothrix* (marine) |
| Cytotoxins | Both fresh and marine cyanobacteria |

### HARMFUL CYANOBACTERIAL BLOOM DYNAMICS

A particularly troublesome aspect of harmful blooms are growing reports of blooms proliferating in previously bloom-free waters experiencing anthropogenic nutrient (most often N and P) enrichment. Historically, the most notorious (i.e., toxin-producing, hypoxia-generating) nuisance algae in freshwaters are cyanobacteria. They include the genera *Anabaena*, *Aphanizomenon*, *Lyngbya*, *Microcystis*, *Nodularia*, and *Oscillatoria* which are traditionally confined to heavily nutrient-enriched impoundments[9,10,13,27,29]. However, global expansion into more-recently eutrophying waters is underway. Examples include the appearance, persistence, and proliferation of toxic heterocystous, $N_2$-fixing genera (*Anabaena*, *Aphanizomenon*, *Cylindrospermopsis*, *Gloeotrichia*, *Nodularia*) and non-$N_2$-fixing genera (*Microcystis*, *Oscillatoria*, *Lyngbya)* in lakes, reservoirs, rivers, and brackish ecosystems throughout Europe, South Africa, Australia, New Zealand, Brazil, Colombia, and Canada. In the U.S., cyanobacterial bloom taxa appear to be expanding in large riverine, estuarine, and coastal ecosystems (e.g., Lake Pontchartrain, Louisiana; St. Johns River Estuary and Florida Bay, Florida; tributaries of the Albemarle-Pamlico Sound System, North Carolina; Potomac River-Chesapeake Bay, Maryland-Virginia). These systems are experiencing increasing agricultural runoff, groundwater, and atmospheric loading of nutrients, especially N and P[37]. Documented toxin and taste/odor-producing genera (*Microcystis*, *Anabaena*, *Aphanizomenon*, *Anabaenopsis*, *Cylindrospermopsis*) are becoming more prevalent and problematic in aquaculture operations, including fresh to brackish catfish, shrimp, striped bass, and euhaline salmon net-pen culture[31]. In the St. Johns River, a riverine-estuarine system draining a large network of eutrophic lakes in central and Northeast Florida, the filamentous heterocystous genus *Cylindrospermopsis*, first reported in the late 1980s[38], has proven to be an aggressive invader of freshwater components of the system. In addition, there are a growing number of reports of more incipient cyanobacterial invasions and outbreaks. Recently, MacGregor et al.[39] provided the first documentation of $N_2$-fixing *Nodularia* spp. in the open waters of Lake Michigan.

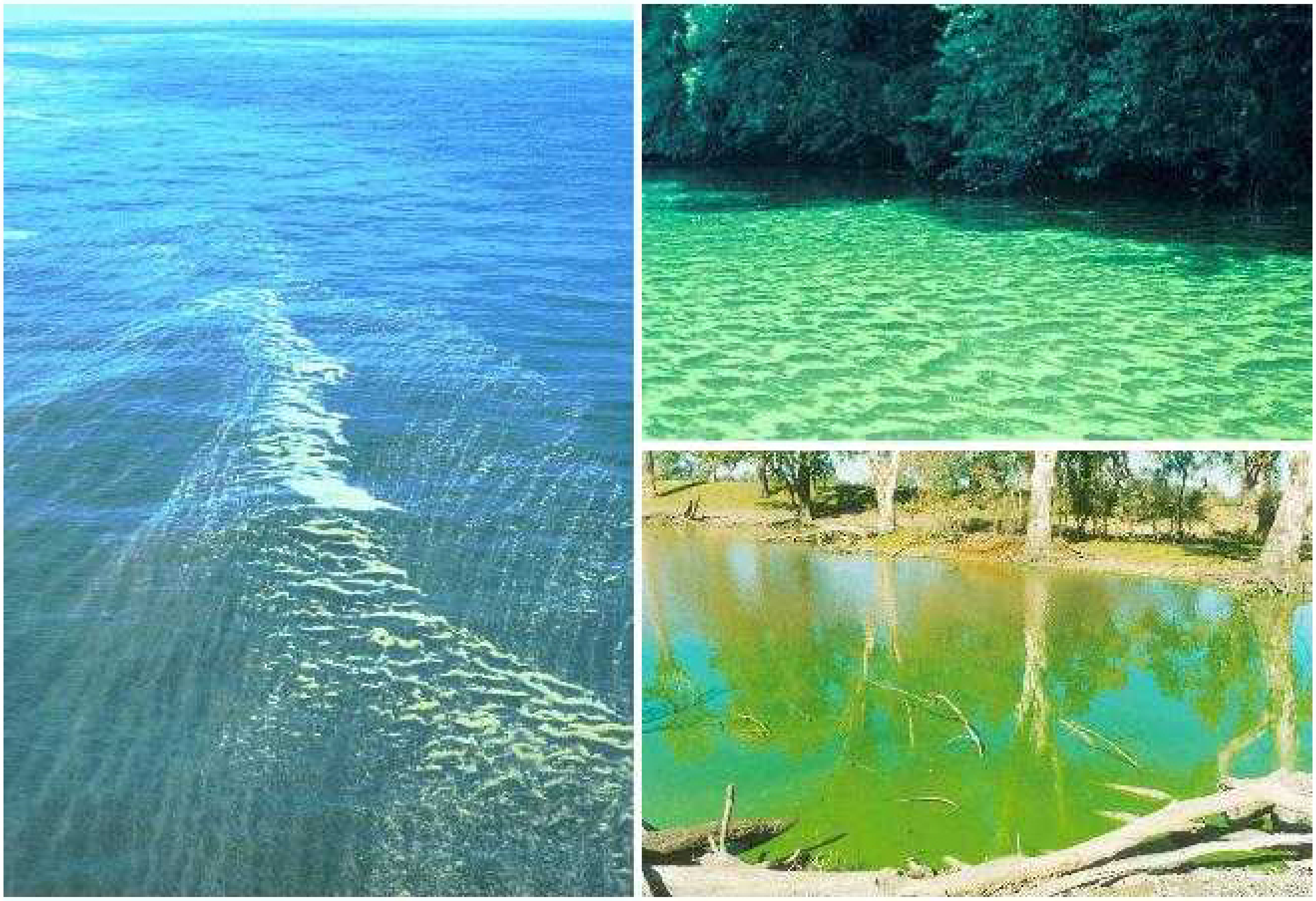

(3A)

**FIGURE 3**. Examples of freshwater cyanobacterial blooms. (A) Upper left, *Nodularia* spp. bloom in the Baltic Sea off the southern coast of Finland; mid-left, *Microcystis aeruginosa* and *Anabaena* spp. bloom in the lower St. Johns River, Florida (courtesy J. Burns); lower left, *Anabaena* spp. bloom in Lake Okaro, New Zealand (North Island); (B) lower right, *Microcystis aeruginosa* bloom in a canal in the Netherlands; mid-right, *Anabaena-Cylindrospermopsis* bloom in Australia (courtesy G. Jones); upper right, *Microcystis-Oscillatoria* bloom in the Neuse River, North Carolina.

What are the environmental factors mediating cyanobacterial bloom expansion? How are these factors related to human activities and hence potential controls and management of these activities? Relevant factors include:

- N and P
- Organic matter
- Iron and trace elements
- Conductivity and salinity
- Turbulence
- Water residence (flushing) times
- Water column vertical stratification and stability
- Interactions with microbes, competitors, and consumers
- Human introduction and displacement of species
- Climate change

Environmental factors implicated in the control of cyanobacterial growth and dominance are listed in Table 4 and conceptually presented in the context of ecosystem function in Fig. 4. Most of these factors are also applicable to other freshwater nuisance algal groups.

### Nitrogen and Phosphorus

On cellular and ecosystem scales, N and P are usually in short supply relative to plant growth requirements[40,41,42,43]. It follows that enrichment of natural waters with one or both

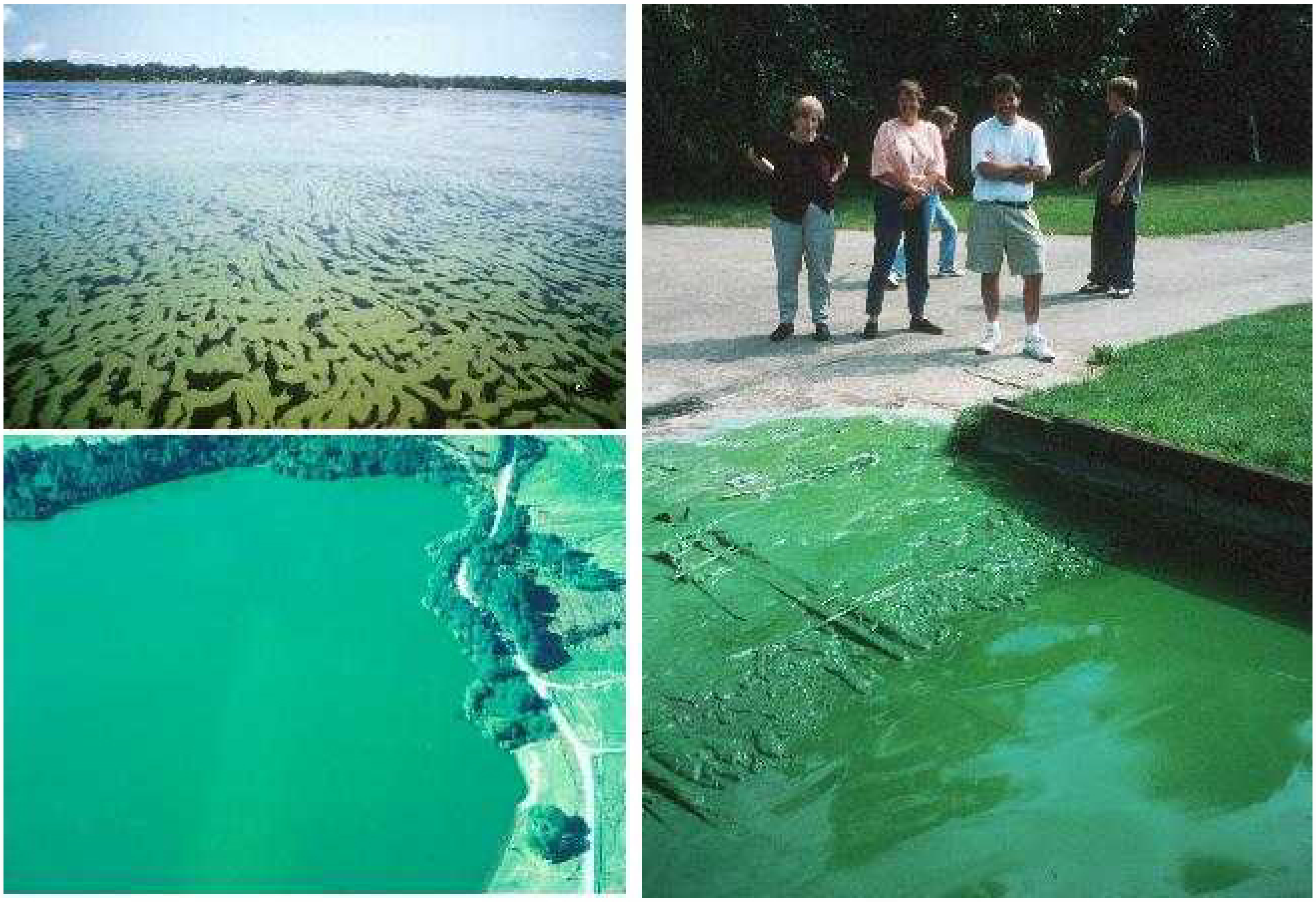

(FIGURE 3B)

of these nutrients stimulates primary productivity. The resultant rate of increase of plant-produced organic matter is called eutrophication; in general, increasing N and P inputs accelerates eutrophication. From an ecosystem perspective, eutrophication can be viewed as a fertilization process. While fertility is essential and desirable from an ecosystem productivity perspective, it is also possible that "too much of a good thing" may result from overfertilization. For example, under conditions of accelerating N and P input, the rate of primary production can exceed the rate at which it is utilized by invertebrate and fish secondary consumers. The "unused" or excess organic matter may then accumulate, initially as planktonic algal blooms and/or massive growths of attached microalgae or macrophytes, then as oxygen-consuming masses of decaying organic matter (detritus). This is the precursor of previously mentioned water quality and health problems, including adverse tastes and odors, toxicity, hypoxia, and anoxia. These initial symptoms of eutrophication can magnify into more serious, long-term water quality and habitat-degradation problems. Hypoxia and anoxia are prime causative agents of shellfish and finfish mortality[44]. They also exert stress on oxygen-requiring higher animals, increasing susceptibility to disease and parasitism.

There is a rich literature pointing to excessive P loading as a chief causative agent of freshwater eutrophication[43,45]. P-driven eutrophication has been strongly implicated in the development of both $N_2$-fixing and non-$N_2$-fixing cyanobacteria. This linkage is particularly strong if affected waters have relatively long residence times (i.e., low rates of flushing), surface water temperatures periodically exceeding 20°C, and vertical temperature stratification (i.e., poorly mixed)[9,10,13,46]. In addition, organic matter-enriched conditions may favor the development and persistence of cyanobacteria[47]. Whether or not $N_2$ fixers dominate the cyanobacterial community depends on N availability as well as the molar ratios of both total and soluble (biologically available) N to P. Waters having N:P <15 (molar) are most susceptible to cyanobacterial dominance[48,49,50]. Conversely, waters having molar N:P ratios in excess of 20 are more likely to be dominated by non-$N_2$-fixing eukaryotic algal taxa[49]. These crite-

**TABLE 3**
**Examples of Recent Environmental and Health Problems Associated with Toxic Cyanobacteria**

| Organism/Toxin | Problem | Reference |
|---|---|---|
| Microcystin (organism unknown) | Net-pen liver disease of maricultured Atlantic Salmon: British Columbia, Canada, and Washington | Carmichael (unpubl.) |
| Microcystin (organism unknown) | Intestinal lesions of maricultured penaeid shrimp: Hawaii, Colombia SA | Carmichael (unpubl.) |
| Microcystin (organism unknown) | Acute lethal liver disease of aquacultured striped bass: California | Carmichael (unpubl.) |
| Microcystin (organism unknown) | Acute lethal liver disease of aquacultured catfish: North Carolina | Zimba et al. (in press) |
| Microcystins (*Microcystis aeruginosa*) | Acute nonlethal toxicity in natural populations of trout and carp: England and Australia | 30, 31 |
| Microcystins | At least 49 human fatalities from use of contaminated municipal water in a hemodialysis clinic: Pernambuco, Brazil | Carmichael et al. (in press) |
| Anatoxin-a, anatoxin-a(s), microcystins, nodularins, and saxitoxins | Continued widespread animal poisonings throughout the world | 14, 31 |
| Cylindrospermopsin (*Cylindrospermopsis raciborskii*) | Cattle poisoning: Queensland, Australia | 142 |
| Cylindrospermopsin (*Cylindrospermopsis raciborskii*) | 148 humans hospitalized with hepatoenteritis in Palm Island, Queensland, Australia due to contaminated water supply | 141 |

ria are most effective in periodically stratified, long residence (> 30 days) systems.

The "N:P rule" is less applicable to highly eutrophic (hypereutrophic) systems in which *both* N and P loadings are very large (i.e., where N and P inputs may exceed the assimilative capacity of the phytoplankton). These systems may periodically contain N:P ratios in excess of 20, but since both N and P are supplied at close to nonlimiting rates, factors other than nutrient limitation (e.g., light, vertical mixing, residence time, conductivity/salinity, trace element availability) may dictate algal community composition and activities. Here, $N_2$ fixation, or diazotrophy, confers little, if any, advantage; hence non-$N_2$-fixing cyanobacteria and other algal taxa will predominate. These conditions favor very high rates of primary production and near-surface bloom accumulation. This, in turn, will reduce clarity and restrict photosynthetically active radiation (PAR; 400 to 700 nm) to the upper few centimeters of the water column. Such conditions provide an ideal niche for the surface-dwelling, bloom-forming, non-$N_2$-fixing cyanobacterial genus *Microcystis*, which invariably dominates in highly eutrophied, stratified ponds, rivers,

**TABLE 4**
**Environmental Factors Influencing Cyanobacterial Growth and Bloom Formation**

| Factor | Impacts and Cyanobacterial Responses |
|---|---|
| **Physical** | |
| Temperature | Temperatures >15°C favor cyanobacterial growth, many species have optima at >20°C. |
| Light | Many bloom genera prefer/tolerate high light, while others are shade-adapted. |
| Turbulence and mixing | Most bloom genera prefer low turbulence over a range of spatial scales, poorly mixed conditions are favorable. |
| Water residence time | Long residence times are preferred by all genera. |
| **Chemical** | |
| Major nutrients (N and P) | Both N and P enrichment favor non-$N_2$-fixing genera. Low N:P ratios (i.e., high P enrichment) favors $N_2$ fixers. |
| Micronutrients (Fe, metals) | Fe required for photosynthesis, $NO^-_3$ utilization, and $N_2$ fixation; evidence for periodic Fe limitation. Other metals (e.g., Cu, Mo, Mn, Zn, Co) required but not limiting. |
| Dissolved inorganic C (DIC) | DIC can limit phytoplankton growth, but cyanobacteria can circumvent this; DIC limitation and high pH may provide competitive advantages to cyanobacterial bloom taxa. |
| Dissolved organic C (DOC) | Many cyanobacterial bloom taxa are capable of utilizing DOC; blooms often flourish in DOC-enriched waters. |
| Salinity | Not restrictive to cyanobacteria *per se*, but some bloom-forming genera (*Anabaena*, *Microcystis*) do not thrive in saline waters. Other genera (*Nodularia*) are salt-tolerant. |
| **Biological** | |
| Grazing | Selective factor, favoring large inedible filamentous and colonial, as well as toxic (to zooplankton) genera |
| Microbial interactions | Consortial cyanobacterial-bacterial interactions may promote growth and bloom formation/persistence. Interactions may be chemically mediated (i.e., role for "toxins"?). Some cyanobacteral-protozoan interactions may also be mutually beneficial. Evidence for viral and bacterial antagonism (i.e., lysis) towards cyanobacteria. However, does not appear to be a common mechanism for bloom control. Cyanobacterial-microbial competition for nutrients exists and may be a competitive mechanism. |
| Symbioses with higher plants and animals | Cyanobacteria are epiphytic/epizoic and form endosymbioses with algae, ferns, and vascular plants. Many are obligate and involve $N_2$-fixing cyanobacterial genera. |

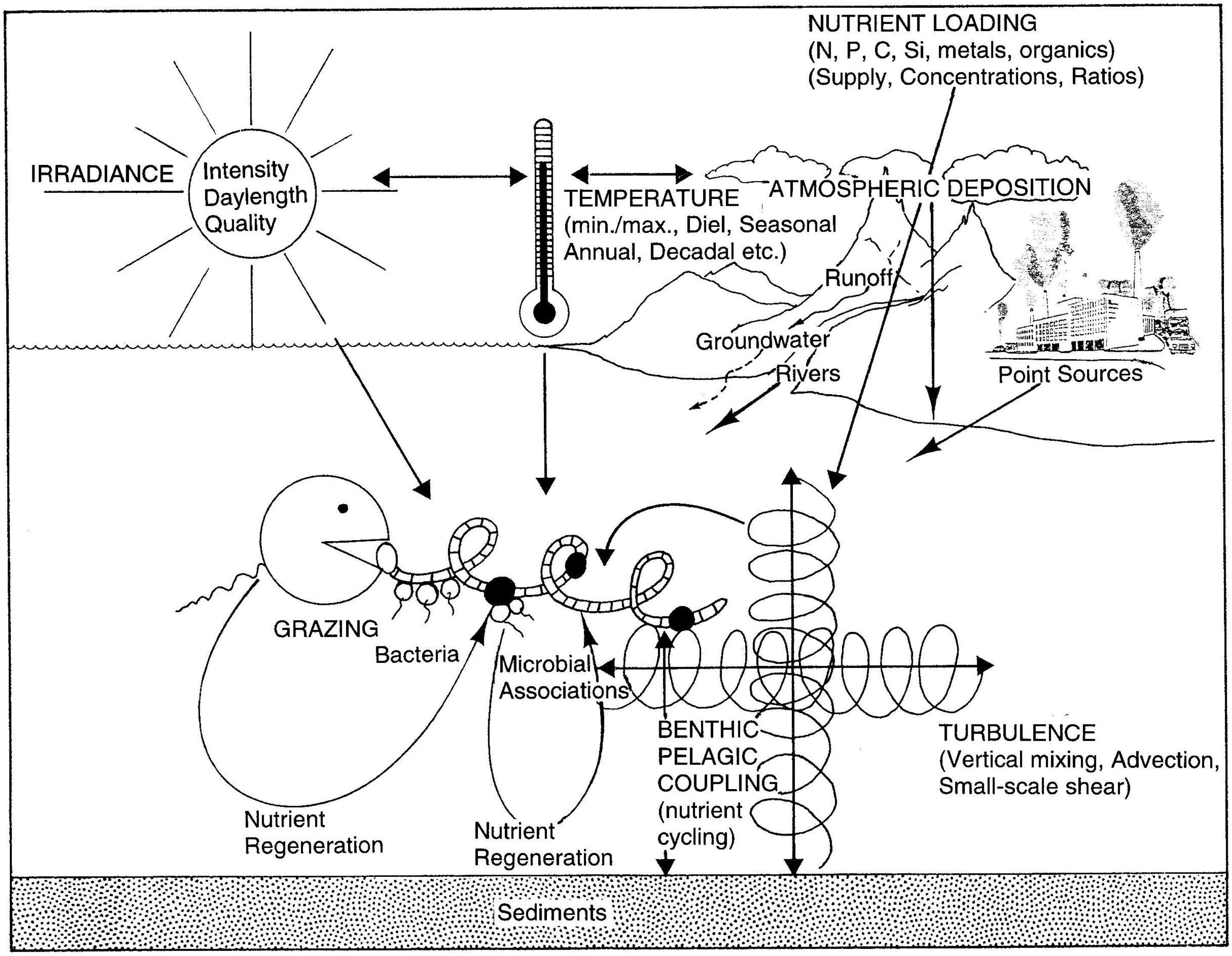

**FIGURE 4.** Conceptual diagram showing the interacting (in time and space) physical, chemical, and biotic environmental controls of cyanobacterial bloom dynamics.

lakes, and reservoirs receiving elevated N *and* P loadings. *Microcystis* blooms are often accompanied by other nondiazotrophic cyanobacterial genera, including *Gomphosphaeria, Oscillatoria,* and *Lyngbya.*

Moderately N- and P-enriched waters frequently support mixed assemblages of diazotrophic cyanobacteria and various nondiazotrophic algal assemblages. In temperate regions, these waters receive either co-occurring or sequential pulses of N and/or P. Typically, late winter and early spring are high precipitation periods, leading to elevated spring nutrient-laden (especially N) nonpoint source (NPS) surface runoff. This favors nondiazotrophic bloom species. In relatively dry summer months, runoff and NPS loading are reduced. During this period, point-source (PS) (e.g., wastewater treatment plants, municipal and industrial sources) and internally generated N and P loads released from hypoxic sediments, become more prominent components of nutrient loading. P enrichment (i.e., declining N:P ratios) occurs during reduced NPS and elevated PS loading during summer months. This scenario is more conducive to the establishment of $N_2$-fixing species[48]. Diazotrophs may be accompanied by nondiazotrophic species, which are able to utilize N fixed and released into the water column by diazotrophs[51]. Buoyancy regulation helps maintain a near-surface existence for both metabolic types. This mechanism is particularly advantageous in highly productive, turbid waters. Mixed assemblages often persist as a bloom "consortium" throughout summer and fall months, until unfavorable physical conditions such as excessive cooling (<15°C) and water column turnover take place. *Anabaena, Aphanizomenon,* and *Microcystis* commonly co-occur under these circumstances.

N-fixing cyanobacterial dominance has been controlled by reducing P inputs, which, in addition to restricting P availability, promotes increased N:P ratios[13,45,49,50]. From logistic and economic perspectives, P input constraints are often the most feasible and least costly short-term nutrient management option[45,49]. In certain cases, P cutbacks can be highly effective on their own (without parallel N removal), because 1) they may reduce total P availability enough to reduce growth of *all* bloom taxa and 2) they may increase N:P ratios sufficiently enough to provide eukaryotic algae a competitive advantage over cyanobacteria. Examples of this scenario include: 1) Lake Washington, Washington, U.S., where reduction of sewage-based P inputs led to profound reversal of eutrophication[52] and 2) reduction of wastewater, agricultural, and industrial P discharges to Lake Erie, which caused a rapid (within only a few years) and sustained decline in nuisance cyanobacterial blooms[43]. In North America and Europe, phosphate detergent bans (starting in the late 1970s) have helped reduce P loads to many rivers and lakes. Reductions in wastewater and agricultural P inputs have also helped. Jointly, these efforts have helped decrease bloom activities in large European and Asian lakes (e.g., Lakes Constance and Lucerne, Germany-Switzerland; Lake Trummen, Sweden; Lago Maggiorre, Italy; Lake Biwa, Japan), and freshwater-brackish lagoonal segments of the Baltic Sea (Latvia, Lithuania, Estonia). Here, a marked reduction of blooms of the $N_2$-fixer *Aphanizomenon* and non-$N_2$-fixer *Microcystis* has been reported[53]. Elsewhere, parallel N and P reductions have been most effective in reducing bloom potentials. In N and P colimited large lake and river systems where large amounts of externally supplied and/or naturally occurring P have enriched sediments, both N and P reductions are required for significant reductions in the size and spatial extent of blooms. Examples include the Florida inland lakes and rivers, Lake Tahoe, Lake Superior, and lakes in the Mississippi River drainage basin.

In contrast to most freshwater systems, primary production in marine waters is generally N-limited, with some estuaries exhibiting N and P colimitation[12,54,55,56,57]. N-enriched estuarine and coastal waters have experienced a recent upsurge in algal blooms[58,59,60,61]. Therefore, management of N inputs has received considerable attention[62,63]. Marine sediments are rich repositories of biogenically deposited P. In shallow water estuarine and near-shore shelf systems, P is efficiently cycled between sediments and the water column, ensuring a readily available source of regenerated P to support productivity. In contrast, marine sediments are generally N-depleted, in large part because microbial denitrification converts substantial amounts of externally supplied N (as nitrate) to relatively inert $N_2$ gas[64]. In addition, sedimentary organic N and ammonium can be nitrified to nitrate, which can also be converted to $N_2$ via denitrification (coupled nitrification-denitrification). $N_2$ fixation rates in these waters appear insufficient to balance denitrification losses[64]. As such, these waters remain chronically N-deficient, with total (soluble plus particulate) N:P ratios ranging from 1 to 5.

In North Carolina's Neuse River estuary, N-driven eutrophication and deteriorating water quality have prompted calls for an N input "cap" and a mandated 30% reduction in N loading (NC DENR Draft Neuse Basin Management Plan, 1997). However, changes in N loading may result in shifts in the ratio of dissolved nitrogen to phosphorus (N:P) loadings and concentrations in the Neuse River Estuary. Alterations in N:P in the river water can have significant impacts on aquatic communities beyond a simple reduction in phytoplankton productivity and biomass. Included are shifts in species composition and possible selection for species adapted to growth in waters with reduced N:P[49,65]. The phytoplankton community could conceivably become dominated by $N_2$-fixing species that may circumvent N limitation imposed by the managed N depletion[51,66]. An assessment of the impact of N-loading reductions on phytoplankton communities is needed to determine if additional controls are necessary to effectively manage nuisance cyanobacterial growth in N-limited estuaries. $N_2$-fixing *Anabaena* and *Cylindrospermopsis* species have been observed in brackish estuarine, lagoonal, and coastal (Baltic Sea) waters[67,68,69], illustrating the *potential* for expansion. However, *Microcystis* does not seem to share this capability[70,71]. In a laboratory study[72], two toxic Baltic Sea *Nodularia* strains[73] were capable of growth and bloom formation in Neuse River Estuary water over a wide range of salinities (0 to >15 psu), again demonstrating the potential for estuarine expansion of nuisance species. These examples indicate that estuarine waters receiving increasing urban and agricultural nutrient inputs are susceptible to cyanobacterial expansion[13].

### Organic Matter

Organic matter content has been mentioned as a possible modulator of cyanobacterial growth and dominance[9,47]. Organic matter (OM) exists in either dissolved (DOM) or particulate (POM) forms; the distinction is based on size fractionation. By designation, DOM is that fraction passing through a glass fiber filter (~0.7 mm pore size)[74]. Most colloidal OM, some bacteria, and virtually all viruses are smaller than this pore size and hence are included in the DOM pool. It is believed that DOM is more directly utilized than POM by microorganisms, because POM degradation (to DOM)

must first occur before this fraction is available. Therefore, DOM content is generally considered the most useful indicator of biologically available OM. DOM may directly stimulate microalgal heterotrophic growth if the organisms possess appropriate uptake and assimilatory enzymes. Many species of cyanobacteria, some dinoflagellates, chrysophytes, and chlorophytes are known to be capable of heterotrophic growth[75]. DOM may also indirectly benefit microalgae by acting as a source of energy and nutrition for closely associated heterotrophic bacteria. Cyanobacteria, in particular, are able to form synergistic interactions with epiphytic heterotrophic bacteria. These associations have been shown to enhance growth of "host" cyanobacteria[76,77]. In some freshwater ecosystems, direct (positive) relationships between trophic state (i.e., oligotrophic systems having low DOM, to eutrophic having high DOM) and cyanobacterial dominance have been observed. Regional examinations however yield no consistent trend, and other coinciding factors, such as pH, alkalinity, and hardness hinder the establishment of simple, direct mechanistic relationships. To further confound matters, Fogg[9] and others[51,78,79] have shown that that elevated DOM may be a result (due to DOM excretion, bacteria and viral lysis, and "sloppy feeding" on cyanobacteria by grazing zooplankton) rather than a cause of cyanobacterial blooms. In summary, while there appear to be direct relationships between DOM enrichment and a preponderance of nuisance blooms (especially cyanobacterial) (Granéli et al., in preparation), it remains unclear what the role(s) of DOM in bloom dynamics are. Well-defined experimental work, utilizing experimental enclosures or whole water bodies will help resolve this perplexing problem.

### Iron and Trace Elements

Iron (Fe) and a suite of trace elements are essential micronutrients for algal growth. Fe is an enzyme cofactor in numerous biochemical pathways. Specifically, enzymes involved in photosynthesis, electron transport, energy transfer, N (specifically nitrate and nitrite) assimilation, and (in the case of cyanobacteria) $N_2$ fixation require Fe. Because of its importance to phytoplankton growth, Fe can play a role as a limiting nutrient in some freshwater systems[80,81]. There are reports of Fe-limited $CO_2$ fixation and growth in lakes and reservoirs varying in trophic state[41], including waters supporting cyanobacterial blooms[82]. Fe limitation has not yet been shown for estuarine ecosystems, possibly because of their dynamic nature (i.e., land-based nutrient runoff and flushing events, periodic hypoxia and anoxia, and close coupling of sediment and water column biogeochemical cycling).

Under oxic conditions typifying surface waters, Fe exists largely in the oxidized ferric ($Fe^{3+}$) form; as insoluble oxides, hydroxides, and carbonates which readily precipitate and deposit in the sediments. Under anoxic conditions, Fe may be released from the sediments as more available reduced $Fe^{2+}$. Therefore, periodic anoxia may enhance Fe availability. Since nuisance blooms may promote anoxic bottom waters, the algae responsible for blooms may at times be controlling their own $Fe^{2+}$ supplies. Bloom-forming cyanobacteria are particularly adept at taking advantage of this positive feedback, since they can migrate between oxygenated surface waters and anoxic bottom waters by altering buoyancy. They are also relatively tolerant of potentially toxic sulfides in the bottom waters[24]. Strongly stratified, poorly flushed systems favor these conditions.

Fe availability is also mediated by naturally occurring DOM that can form metal chelates. Colored humic and fulvic compounds capable of chelating Fe have received particular attention because they may be plentiful in some systems. In natural waters, the availability of Fe is likely to be mediated by multiple factors, including the forms and concentrations of Fe, organic matter content, irradiance, and the presence of other metals, which may compete with Fe for organic ligands and anions[83].

Bloom-forming cyanobacteria produce potent siderophore (hydroxamate) chelators capable of sequestering Fe at low ambient concentrations[84]. This may provide a competitive advantage over eukaryotic phytoplankton, especially when Fe availability is restricted[85]. However, it appears that Fe limitation is less common than N or P limitation in freshwater. Therefore, relative to the impacts of excessive P loading (and low N:P ratios), Fe limitation most likely plays a secondary role in determining the distributions and magnitudes of cyanobacterial blooms.

Cyanobacteria require a suite of trace metals for various metabolic, growth, and reproductive processes. Manganese, cobalt, copper, molybdenum and zinc are most frequently mentioned[86]. Photosynthesis and $N_2$ fixation require manganese, zinc, cobalt, copper, and molybdenum for synthesis and function. In the case of copper (Cu), there exists a fine line between potentially limiting and toxic ($>\mu M$) concentrations. The biologically available form ($Cu^{2+}$), may be strongly bound by organic ligands, such as humic and fulvic acids. Bioassays of the cyanobacteria-dominated (*Anabaena, Aphanizomenon, Microcystis*) Chowan River, North Carolina, a coastal river enriched with humic and fulvic acids from surrounding swamps, wetlands, and pulp mill effluent, showed that Cu additions ($CuSO_4$; $<1\ \mu M$) stimulated photosynthetic $CO_2$ fixation relative to untreated controls during a period of high humic discharge[87] (Fig. 5). Cu stimulation of productivity was not observed during low humic discharge, indicating that in the presence of elevated humic binding, limitation by Cu and possibly other chelatable metals (Fe) may exist in nature.

Molybdenum (Mo) is a cofactor of nitrogenase, the enzyme complex mediating $N_2$ fixation. Its availability

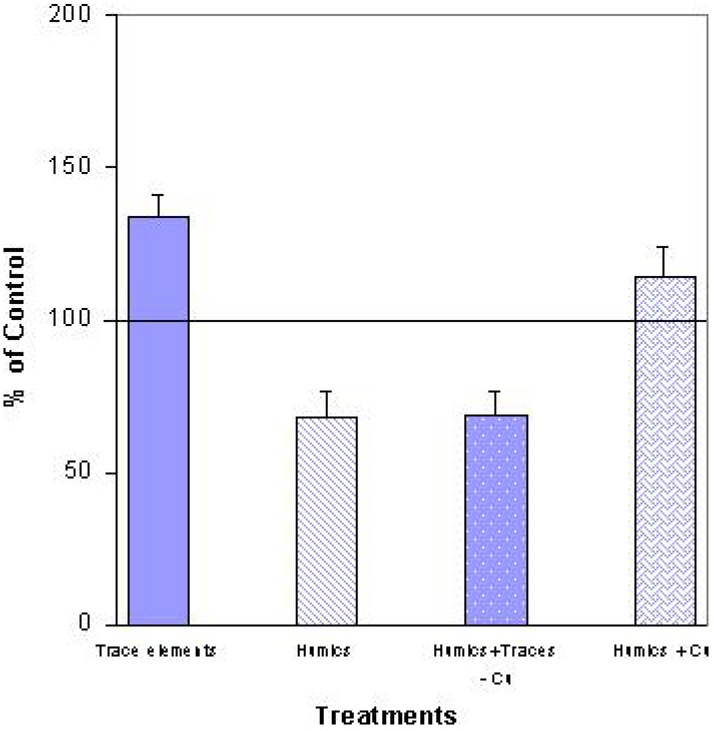

**FIGURE 5**. Results from an *in situ* bioassay of Chowan River (North Carolina) water. Photosynthetic growth of the resident phytoplankton community was assessed by measuring radiolabeled $CO_2$ uptake for 48 h. Controls were untreated samples and treatments were compared to controls as percent of control $CO_2$ uptake. Treatments included: trace element mix (Mn, Zn, Fe, Mo, B, Mg, Co), humic acid (from swampwater and pulp mill effluent entering the river) addition (at naturally occurring dilutions), with and without trace element mix minus Cu, and humic acid with only Cu added. All trace elements were added at 0.5 mM.

could therefore play a regulatory role in diazotrophic cyanobacteria. Howarth and Cole[88] proposed that the relatively high (>20 mM) concentrations of sulfate ($SO_4^{2-}$), a structural analogue of molybdate ($MoO_4^{2-}$, the dominant form of molybdenum in seawater), could competitively (via the uptake process) inhibit $N_2$ fixation, thereby limiting this process. Competitive inhibition of $MoO_4^{2-}$ uptake by high $SO_4^{2-}$ concentrations was shown by Cole et al.[89]. However, $MoO_4^{2-}$ is highly soluble in seawater where concentrations of ~100 µM are commonly found. Ter Steeg et al.[90] and Paulsen et al.[91] showed that, despite the potential for $SO_4^{2-}$ competition, Mo availability was maintained at concentrations much lower than 100 µM. In coastal and pelagic ocean (W. Atlantic) waters, $N_2$-fixing potentials of marine diazotrophs appear unaffected by this competition[91]. Furthermore, in freshwater, $SO_4^{2-}$ concentrations are much lower (<10%) than seawater. Most likely, the small cellular Mo requirements for $N_2$ fixation are met through reduced but sufficient uptake and storage. In addition, there are "alternative" non-Mo-requiring nitrogenases in bacterial and cyanobacterial diazotrophs[92]. If such microbes are broadly distributed in nature, it would represent a mechanism by which Mo limitation could be circumvented.

Trace metal addition experiments with natural phytoplankton populations indicate that in most freshwater systems availability meets growth demands. Therefore, it is unlikely that trace metal limitation is a widespread modulator of growth and bloom potentials. Trace metals may, however play synergistic roles with major nutrients (N, P) in determining phytoplankton competitive interactions, activities, and composition.

### Conductivity and Salinity

Salinity and ionic strength (conductivity) of waters have been mentioned as a potential regulators of $CO_2$ and $N_2$ fixation of bloom-forming cyanobacteria[67,68,69,70,71]. This may be true for some strictly freshwater species. However, recent work on the bloom-forming, $N_2$-fixing nuisance genus *Nodularia* argue against making generalities[68,69,72]. Two *Nodularia* species recently isolated from the Baltic Sea (original salinities ~4 to 15 psu) are able to grow in the laboratory from near 0 to >30 psu salinity (Table 5). Moreover, in laboratory experiments, these species were able to grow in Neuse River Estuary (North Carolina) water throughout these salinities, indicating a *potential* for expanding beyond its current geographic range[71]. The only nutrient requirement for expansion in Neuse estuary was adequate P supply. Similar results have recently been obtained for other common heterocystous diazotrophs, including *Anabaena* and *Anabaenopsis* (Table 5). On the other hand, the aggressively expanding (in eutrophying tropical and subtropical freshwater systems) heterocystous cyanobacterium *Cylindrospermopsis raciborskii*, and the ubiquitous non-$N_2$-fixing bloom-former *Microcystis aeruginosa,* cannot tolerate salinities in excess of 2 psu (Table 5). Among many other diazotrophic genera, salinity does not appear to be a barrier to growth and reproduction. For example the epiphytic and epibenthic $N_2$-fixing genera (e.g., *Calothrix, Nostoc, Scytonema*) are present in estuarine and coastal ecosystems worldwide[93,94]. Cyanobacterial $N_2$ fixation in some freshwater species may be particularly susceptible to osmotic stress[95], and organisms unable to adjust by the production of compensatory factors show inhibition of activity at increasing salt concentrations[96,97]. Cyanobacteria introduced from soils or freshwater into an estuarine environment may not be able to compensate for increasing salinities and osmotic stress[97,98]. Indigenous populations are often able to adjust to varying salinities by the production of compatible osmolytes[98]. There has been relatively little consideration of this as a factor regulating estuarine $N_2$ fixation.

### Turbulence

Turbulence plays a fundamentally important role in regulating phytoplankton bloom dynamics. Cyanobacteria are especially sensitive to water column stability, including vertical stratification[2]. The ramifications of ecosystem-level physical constraints such as residence time or flushing rate have been alluded to already[2,6,10]. Combined with mesoscale circulation, transport, and vertical mixing controls, small-scale turbulence, or shear, is of central importance in determining growth potentials and dominance among $N_2$-fixing and non-$N_2$-fixing cyanobacteria[10,13,35]. Both the magnitude and duration of turbulence alter phytoplankton growth rates and structural integrity[99,100]. Among cyanobacteria, nondisruptive, low-level turbulence is known to promote localized

**TABLE 5**
**Salinity Tolerance of Bloom-Forming Cyanobacteria**

| | Genus/Species | Salinity Limits for Growth (PSU) | Reference |
|---|---|---|---|
| $N_2$ fixing | *Anabaena aphanizomenoides* | 0 – 15 | Moisander et al. (in preparation) |
| | *Anabaena torulosa* | 0 – >14.6 | 67 |
| | *Anabaenopsis* | 0 – >20 | Moisander et al. (in preparation) |
| | *Aphanizomenon* | 0 – 5 | 68 |
| | *Cylindrospermopsis* | 0 – 4 | Moisander et al. (in preparation) |
| | *Nodularia* | 5 – >30 | 68 |
| | | 0 – 35 | 67 |
| Non-$N_2$ fixing | *Microcystis* | 0 – 2 | 69, 70 |
| | *Oscillatoria* | 0 –>30 | 24, 92 |

"phycosphere" nutrient cycling, alleviate certain forms of nutrient limitation (DIC, $PO_4^{3-}$, trace metals), and enhance growth[24,101]. Gently stirred cultures of bloom-forming heterocystous genera (including *Anabaena, Aphanizomenon, Nostoc,* and *Gloeotrichia)* frequently grow faster than static cultures[9,24]. Increases in turbulence either as stirring or shaking (Table 6) or more well-defined small-scale shear (Table 7) can, however, inhibit photosynthetic and $N_2$-fixing activities and growth, with excessive turbulence causing disaggregation, cell and filament damage, and rapid death "crashes" among diverse colonial genera in culture and in nature[51]. $N_2$ fixation, photosynthetic and growth performance in these genera often rely on mutually beneficial microbial (heterotrophic-autotrophic) consortial interactions with host cyanobacteria. Turbulence can disrupt consortial interactions, and thus act as a negative growth factor.

Cellular-scale turbulence, or shear, can disrupt filaments of heterocystous cyanobacteria. The narrow junctions between heterocysts and adjacent vegetative cells are prone to disruption and filament breakage[102,103]. Filament breakage at the heterocyst-vegatative cell junction cause heterocysts to lose their ability to fix $N_2$ and maintain $O_2$-free conditions, as witnessed by absence of tetrazolium salt (TTC) reduction[104].

Larger-scale (mesoscale) wind and tide-induced mixing affects vertical and horizontal distributions of cyanobacterial bloom taxa[105]. Most bloom genera can regulate buoyancy by varying the density and size of intracellular gas vesicles[10,106]. In turbulent waters, the ability to maintain optimal vertical positioning can be overcome by mixing[107]. Physical forcing of this sort is considered to play an important role in shaping phytoplankton resource (light, nutrient) competition, community composition, and succession[10,13]. Recently, we[51,108,109] showed that levels of shear stress representative of exposed large lake, estuarine, and coastal surface waters might control $N_2$ fixation in bloom-forming genera (*Anabaena, Nodularia*) (Moisander et al., in review), thus representing a potential barrier to their expansion. The observed negative impacts could be due to 1) breakage of cyanobacterial filaments and 2) disruption of growth-promoting phycosphere consortial bacterial-cyanobacterial associations[72,110].

### Genetic Constraints on Cyanobacterial Expansion

Current research has shown broad *potential* for expansion into eutrophying mesohaline and euhaline waters among the genetically diverse array of $N_2$-fixing and nonfixing cyanobacteria. Molecular methods are now being employed in the detection of nuisance cyanobacterial bloom formers[111,112]. Analyzing the DNA present in a water sample is a more rapid method of detection and identification than traditional microscopy. Once the DNA from the phytoplank-

**TABLE 6**
**Impact of Stirring Speed and Resultant Filament Breakage on Chlorophyll *a*-Specific $N_2$ Fixation (Nitrogenase Activity as Acetylene Reduction) in *Anabaena oscillarioides***

| Stirring Speed (rpm) | Nitrogenase Activity (µmol $C_2H_4$ mg Chl $a^{-1}$ $h^{-1}$) |
|---|---|
| 50 | 7.5 ± 0.5 |
| 100 | 7.2 ± 0.7 |
| 250 | 5.8 ± 1.2 |
| 400* | 3.2 ± 0.5 |
| 550* | 2.4 ± 0.7 |
| 750* | 1.2 ± 0.4 |

*Note:* Filaments were exposed to a range of stirring speeds using a magnetic stirring table. Controls are slowly stirred (50 rpm) conditions. When observed, filament breakage is indicated as *. Controls and treatments were grown under N-limited conditions (Chu-10 medium minus N) under continuous illumination (200 µmol $m^{-2}$ $sec^{-1}$) at 25°C. Standard errors among triplicate samples are shown (adapted from Paerl and Zehr[112]).

**TABLE 7**
**Mean $N_2$- and $CO_2$-Fixation Rates as a Function of Exposure to Small-Scale Laminar Shear in Two Cultured Species of *Anabaena***

| | *Anabaena oscillarioides* | | *Anabaena circinalis* | |
|---|---|---|---|---|
| | $N_2$ Fixation (nmol $C_2H_4$ mg Chl*a*$^{-1}$ h$^{-1}$) | $CO_2$ Fixation (mg C mg Chl*a*$^{-1}$ h$^{-1}$) | $N_2$ Fixation (nmol $C_2H_4$ mg Chl*a*$^{-1}$ h$^{-1}$) | $CO_2$ Fixation (mg C mg Chl*a*$^{-1}$ h$^{-1}$) |
| Control | 4.98 (± 1.8) | 2.31 (± 0.5) | 8.52 (± 1.2) | 1.75 (± 0.6) |
| Low Shear | 4.26 (± 1.5) | 2.03 (± 0.4) | 7.27 (± 1.1) | 1.65 (± 0.4) |
| High Shear | 3.29 (± 1.5) | 1.34 (± 0.5) | 4.29 (± 0.9) | 1.32 (± 0.3) |

*Note:* Shear was exerted by placing *Anabaena* populations in Couette chambers, which are capable of exerting specific, highly controllable shear fields. Shear mimicked wind-driven shear conditions in estuarine surface waters. It is expressed as low (0.93 to 5 rpm in the chamber) or high (15 to 36 rpm). Results are compared to Controls, in which no shear (i.e., no chamber rotation) was exerted. Triplicate $N_2$ fixation (acetylene reduction assay) and $CO_2$ fixation ($^{14}CO_2$ fixation) determinations were based on 2-h incubations following 4-h exposure to specific shear fields. Values in parentheses indicate standard error of the mean. Data adapted from Kucera[109].

ton in a sample is isolated, the genetic sequences of particular genes are determined and the cyanobacteria are identified based upon the comparison of these gene sequences with a database of previously identified genetic sequences[111,112]. These sequences are compared in a phylogenetic tree, which is used to visualize the degree of similarity between organisms based on genetic data. Organisms that are more closely related cluster together on the branches of the tree. All cyanobacteria contain the 16S rRNA gene, a structural gene present in prokaryotes, so this gene was used for comparing both $N_2$-fixing and non-$N_2$-fixing cyanobacteria (Fig. 6). Diazotrophs ($N_2$ fixers) contain the *nifH* gene, which encodes one of the proteins that makes up the nitrogenase enzyme that is necessary for $N_2$ fixation. *NifH* sequences are used to study relatedness of specific $N_2$-fixing cyanobacteria, but can also be used to identify noncyanobacterial heterotrophic diazotrophs. Heterocystous cyanobacteria consistently cluster closely together in *nifH* phylogenetic trees, demonstrating their high degree of genetic similarity (Fig. 7). Diazotrophs can be identified in this manner, regardless of whether they are actively fixing $N_2$.

Using these molecular methods, the genetic potential (i.e., presence of *nifH*) for $N_2$ fixation was identified in the Neuse River, North Carolina over both spatial and temporal scales. The presence of *nifH* was identified in both surface and bottom waters at stations ranging from the freshwater upstream portion of the river down to the mesohaline mouth where it empties into Pamlico Sound (Dyble, in preparation). *NifH* was also present throughout most of the year, even at times when cyanobacteria were not numerically dominant in the water column. Sequencing some of these *nifH* genes revealed genetic sequences that were most similar to heterocystous cyanobacteria (Dyble, in preparation). Thus, the use of molecular methods has revealed that cyanobacterial $N_2$ fixers are present in this river system, even at higher salinities and colder temperatures. However, for this genetic potential to be expressed into active $N_2$ fixation requires the environmental conditions described above to be satisfied..

### Biotic Interactions

#### *Interactions with Other Microbes*

Bloom-forming cyanobacteria form close associations with other microorganisms and higher organisms (Table 8, Fig. 8). Cyanobacterial-microbial associations are common among bloom-forming genera, including *Anabaena, Aphanizomenon, Microcystis,* and *Nodularia*[113,114,115,116,117]. Associated microorganisms include eubacteria[76,78,107,115,118], fungi[76,116,117], phytoflagellates[119], and ciliated and amoeboid protozoans[120,121]. Most associations occur within and around colonies, aggregates of filaments, and within the fibrillar-mucilaginous sheaths, capsules, and exuded polymers[76,116,118,122] (Fig. 8).

Specific associations, where certain microbial populations exclusively attach to distinct cyanobacterial host cells (i.e., akinetes and heterocysts), have also been observed (Fig. 8). The intensity and specificity of these associations

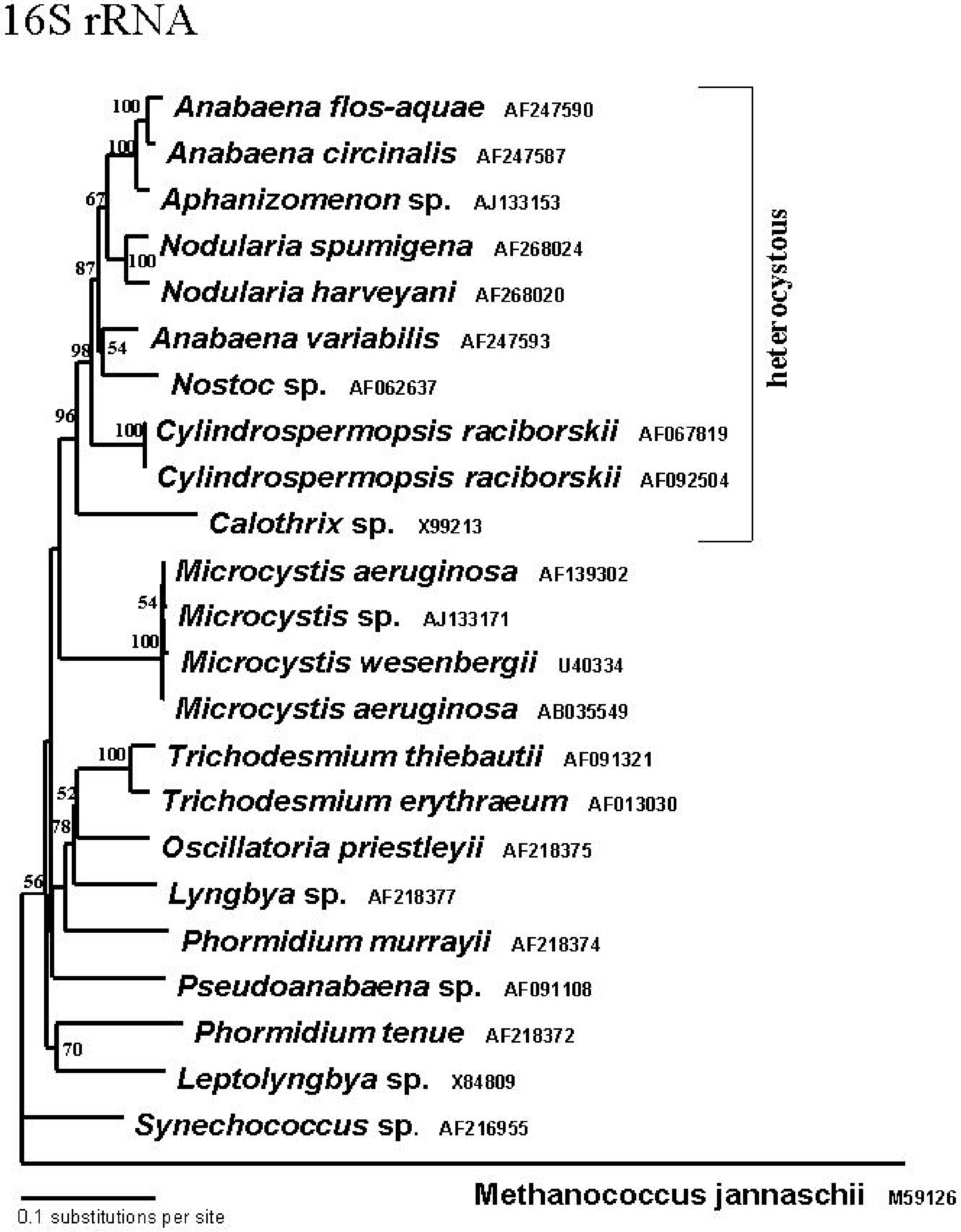

**FIGURE 6.** Cyanobacterial phylogenetic tree based upon 16S rRNA sequences. This tree was constructed by the neighbor-joining method and bootstrap values >50% are given above or beside the corresponding nodes.

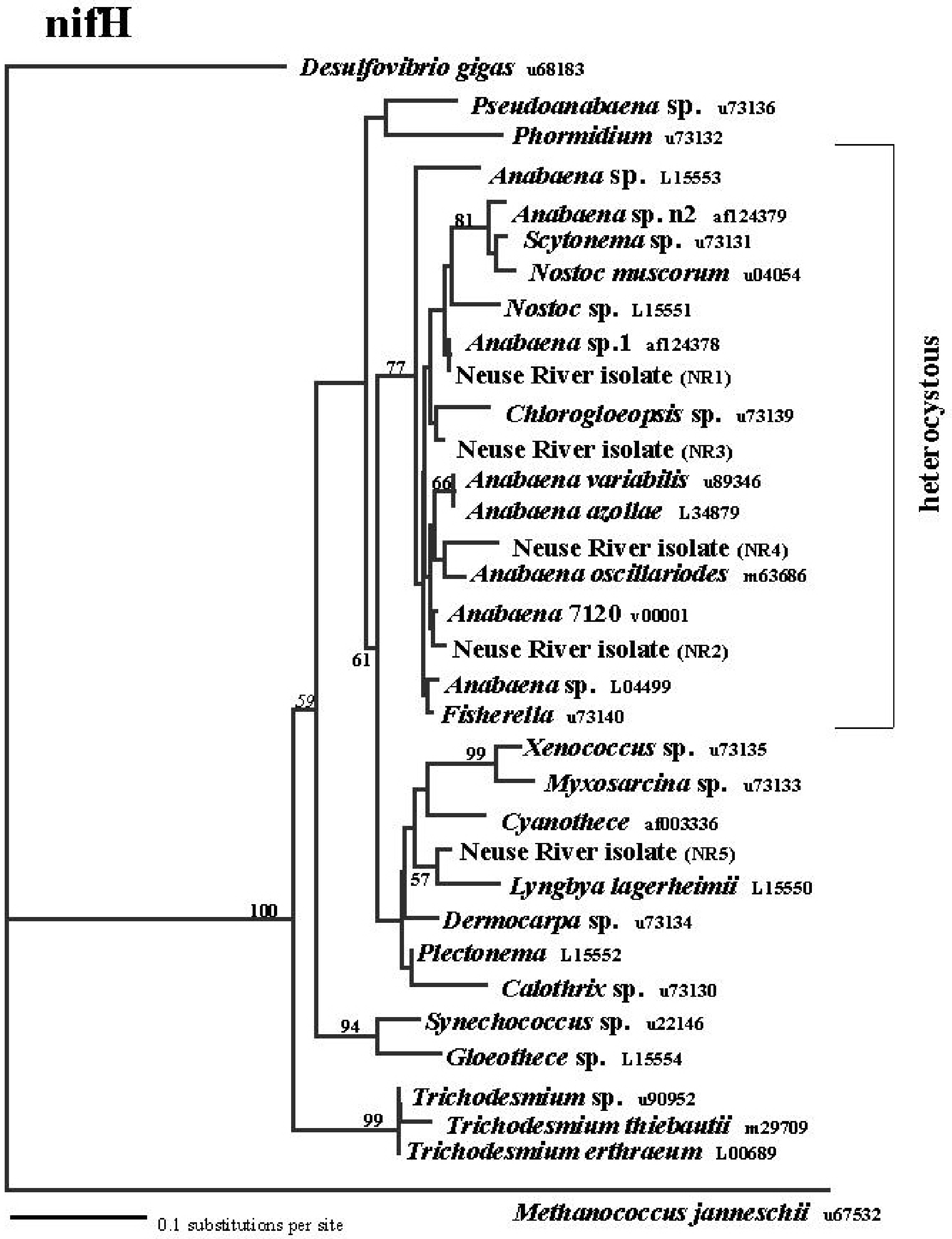

**FIGURE 7**. Cyanobacterial phylogenetic tree based upon *nifH* sequences. This tree was constructed by the neighbor-joining method and bootstrap values >50% are given above or beside the corresponding nodes

**TABLE 8**
**Some Examples of Cyanobacterial Associations with Other Microorganisms, Higher Plants and Animals in Aquatic Ecosystems**

| Association | Cyanobacterial Genera Involved |
|---|---|
| Heterotrophic bacteria (as epiphytes) | *Anabaena, Aphanizomenon, Gloeotrichia, Microcystis, Nodularia, Nostoc* |
| Eukaryotic alga (as endosymbionts) | |
| Marine diatoms (*Rhizosolenia*) | *Richelia* |
| Freshwater diatoms (*Rhopalodia*) | *Synechococcus* |
| Fungi (lichens; as endosymbionts) | *Nostoc, Scytonema* |
| Bryophytes (as endosymbionts in *Blasia*) | *Nostoc* |
| Freshwater Ferns (as endosymbionts in *Azolla*) | *Anabaena* |
| Gymnosperms (as endosymbionts in *Gunnera*) | *Nostoc* |
| Protozoans (epizoic) | *Anabaena, Microcystis, Nodularia* |
| Protochordates (as endosymbionts) | *Prochloron** |
| Marine Gastropods (as endosymbionts) | *Synechococcus* |
| Mammals (in polar bear hairs) | *Synechococcus* |

* Members of the phylum Prochlorophyta.

can vary dramatically[76,107,116]. During the initiation and proliferation of *Anabaena oscillarioides* blooms, when maximum biomass-specific rates of photosynthesis and $N_2$ fixation were observed, an association between the heterotrophic bacterium (*Pseudomonas aeruginosa*) and the heterocysts prevailed[123] (Fig. 8). Virtually all (~98%) of *P. aeruginosa* cells were found attached to heterocysts as opposed to non-$N_2$-fixing vegetative cells of *Anabaena* filaments[113,117,124]. The intensity and frequency of *P. aeruginosa* attachment to heterocysts exhibited a diel pattern[113], with maximum attachment occurring near midday. *P. aeruginosa* was chemotactically attracted to *A. oscillarioides* heterocysts and N-containing amino acids[119,123].

Cyanobacteria excrete organic compounds[79,125], including organic and amino acids, peptides, alkaloids, carbohydrates, and lipopolysaccharides[79,125,126,127,128]. Diverse excretion products chemotactically attract and support the growth of phycosphere-associated bacteria[124,129]. In $N_2$ fixation and fate experiments, some of the $^{15}$N-labeled $N_2$ fixed by host *A. oscillarioides* was rapidly transferred to heterocyst-associated *P. aeruginosa*[130]. Axenic isolates of *A. oscillarioides* exhibited optimal growth and $N_2$-fixation rates when reinoculated with *P. aeruginosa*[113] (Fig. 9). When released in axenic *A. oscillarioides* cultures, *P. aeruginosa* re-established the heterocyst-specific association.

These findings suggest close metabolic coupling and mutually beneficial relationships. Culturable cyano-bacterial bloom species, including strains of *Microcystis aeruginosa* and an *Aphanizomenon flos-aquae* strain from North Carolina coastal rivers have not been successfully grown free of bacteria[76]. Among other cyanobacterial genera (e.g., *Oscillatoria, Lyngbya, Nodularia*), bacterized strains revealed higher growth rates and were easier to maintain in culture than axenic strains. Gibson and Smith[131] reported that both axenic *Oscillatoria redekei* and *O. agardhii* isolates "always appeared to grow better in the presence of contaminant heterotrophic bacteria". Similar findings were reported by Allen[132], Gorham[133], Staub[134], Meffert[135], Herbst and Overbeck[136], Caldwell[137], and Lehtimäki et al.[69]

The ecological mechanisms underlying cyanobacterial-bacterial synergism continue to be subjects of investigation. Proposed mutually beneficial mechanisms include exchange of metabolites and growth factors as well as detoxifying roles of associated bacteria. Kuentzel[114] and Lange[78] suggested that

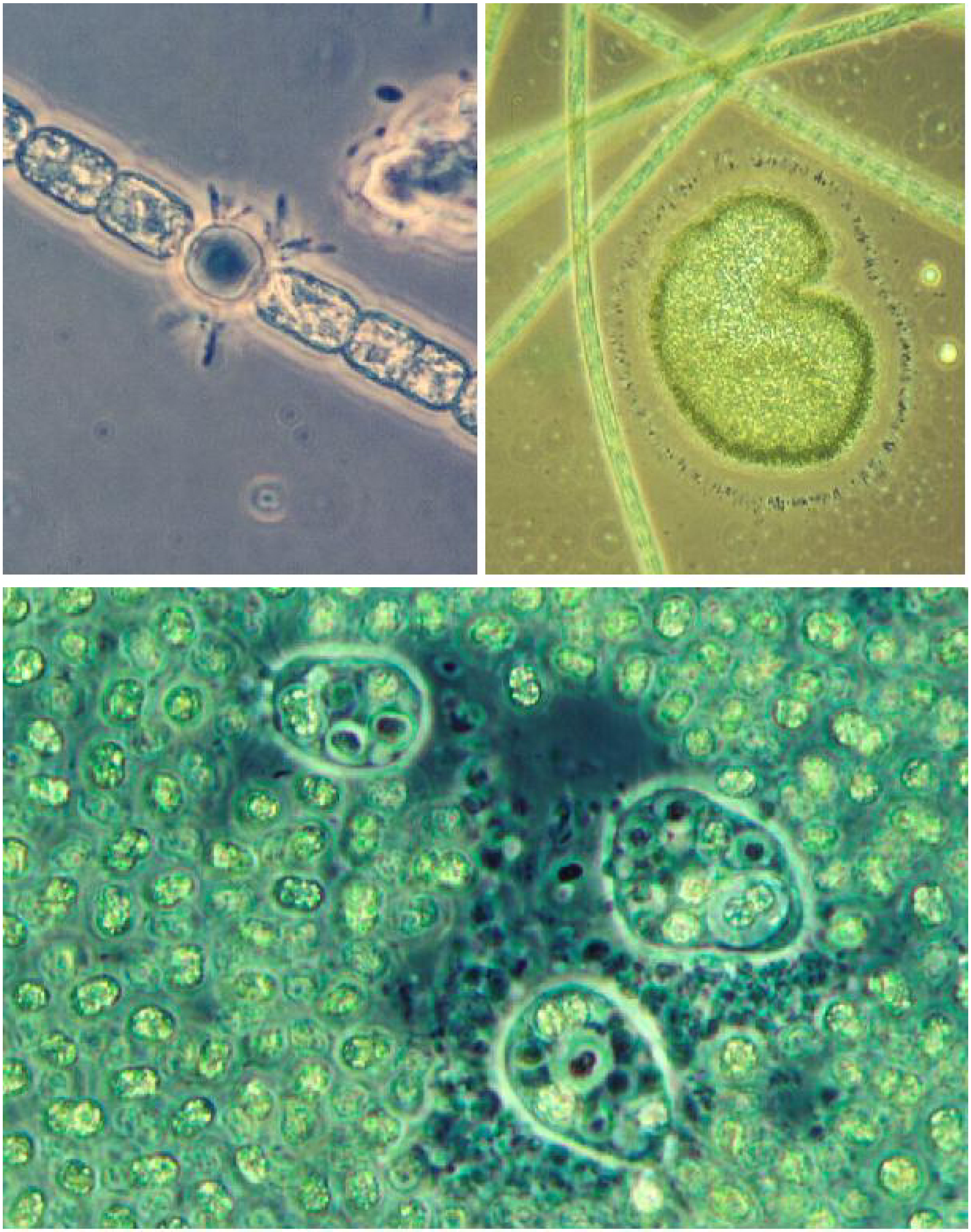

**FIGURE 8**. Examples of microbial associations with bloom-forming cyanobacteria. Upper left, heterotrophic bacteria specifically associated with the nitrogen-fixing cells, or heterocysts, of *Anabaena oscillarioides*. *A. oscillarioides* was originally isolated from the Waikato River, New Zealand (see References 112, 118, and 122 for the functional significance of this association); upper right, a bacterial "halo" commonly observed around colonial cyanobacteria (in this case the "host" cyanobacterium is *Gomphosphaeria*, freshly collected from the Neuse River, North Carolina); lower, amoeboid protozoans "grazing" inside a *Microcystis aeruginosa* colony freshly collected from the Neuse River, NC. Note the ingested *Microcystis* cells in the protozoans. (For details of this association, see Reference 120.)

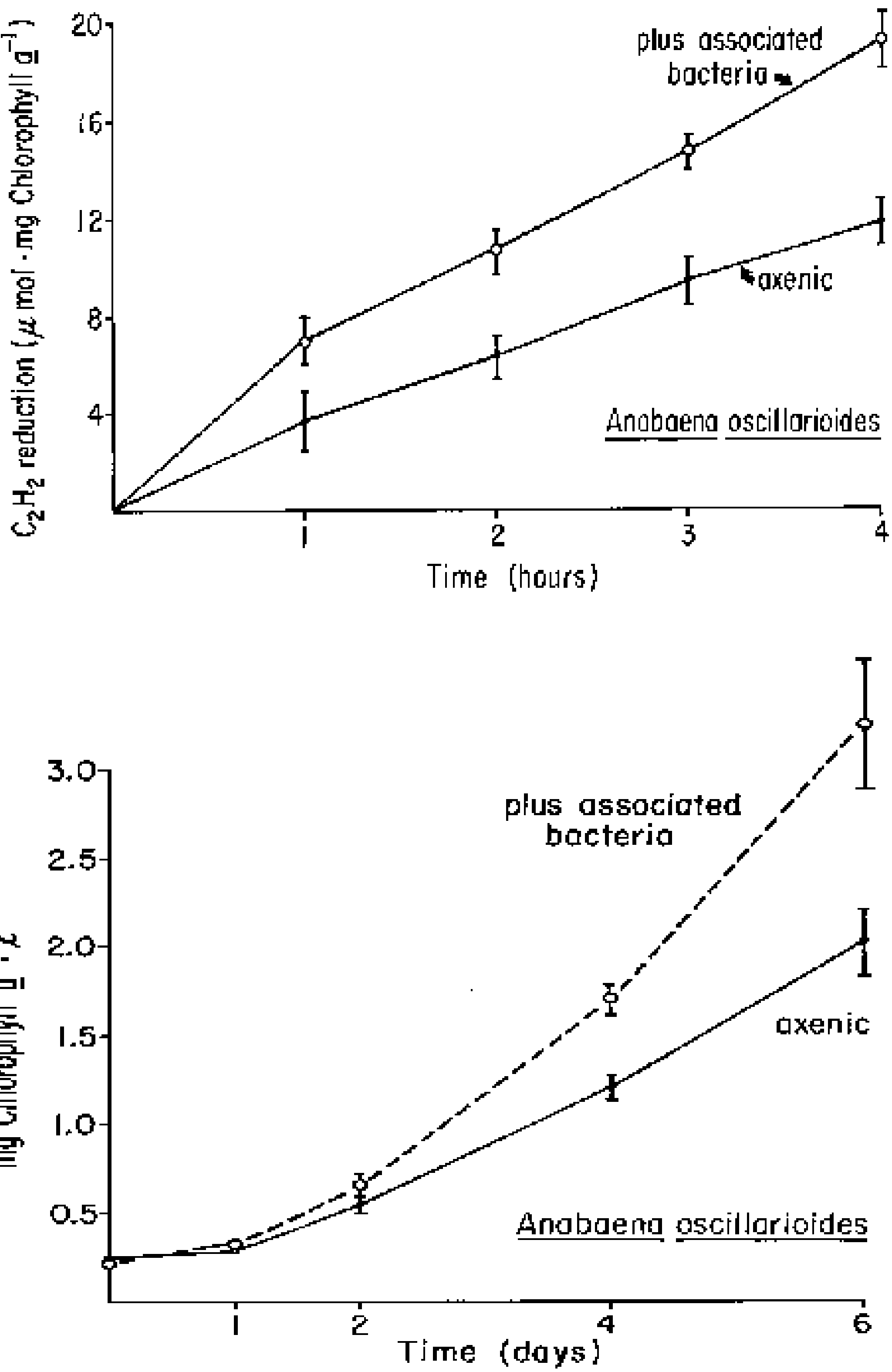

**FIGURE 9**. Nitrogen fixation and growth characteristics of *Anabaena oscillarioides* (same strain as shown in Fig. 8) grown under bacteria-free (axenic) and bacteria-associated conditions. Cultures were maintained on N-free Chu-10 media under 14 h illuminated/10 h dark conditions. Error bars indicate the standard deviation of triplicate measurements at each sampling point. Upper, nitrogen fixation (nitrogenase activity), measured as acetylene reduction per mg chlorophyll *a* per hour; lower, growth measured as mg chlorophyll *a* per liter. (For details, see References 118 and 120.)

$CO_2$ recycling by bacteria proved essential for optimal growth of cyanobacteria under inorganic carbon limited growth, which can occur in dense blooms[138]. Lange[78] demonstrated that the addition of metabolizable carbohydrates led to enhanced cyanobacterial growth, especially under inorganic carbon limited conditions. He attributed such photosynthetic stimulation to rapid mineralization followed by $CO_2$ release among associated bacteria. Schiefer and Caldwell[139], using $CO_2$-limited continuous cultures of *Anabaena* sp. and the epiphytic heterotrophic bacterium *Zoogloea* sp., demonstrated that $CO_2$ recycling by *Zoogloea* sp. optimized photosynthetic performance and growth of *Anabaena* sp. Paerl and Kellar[110] and Kellar and Paerl[140] showed that bacteria commonly associated with heterocysts of bloom-forming *Anabaena* and *Aphanizomenon* (Fig. 8) enhanced $N_2$-fixation potentials, when compared to axenic conditions (Fig. 9). Similarly, Lupton and Marshall[141] confirmed a positive impact of associated bacteria on *Anabaena* sp. growth. Associated bacteria may be instrumental in locally decreasing $O_2$ tension near cyanobacterial cells and filaments harboring biochemical processes (photosynthesis, $N_2$ fixation) that are $O_2$-sensitive[113].

Specific growth factors (e.g., vitamins) produced by associated bacteria may aid cyanobacterial growth. Associated bacteria may also detoxify cyanobacterial extracellular metal (Fe) chelates potentially autotoxic to host cyanobacteria[130].

Certain bloom-forming cyanobacterial strains produce peptides and alkaloids known to have potent cyto-, hepato-, and neurotoxic effects on mammals, fish, birds, and invertebrates residing in bloom-infested waters[15,31,142,143]. From ecological and evolutionary perspectives, it is difficult to rationalize such "toxin" production, since affected organisms are not natural consumers of cyanobacteria. More likely, mammalian and fish toxicity is coincidental, and because of higher animal and human health concerns, defined in an anthropomorphic rather than ecological context. In aquatic ecosystems, where microbial competition for dilute organic and inorganic compounds is intense[104,144], cyanobacterial toxin production may serve to select for and maintain mutualistic and symbiotic microbial relationships[145]. Toxins may deter protozoan and metazoan grazing, viral and bacterial lysis, while promoting mutually beneficial microbial associations. In this case, cyanobacterial toxins selectively create and condition microenvironments surrounding filaments and colonies. Understanding the means by which this form of "microbial gardening" is regulated by environmental factors (and their interactions) (including nutrient, light, temperature, salinity, desiccation, and other forms of stress) may yield mechanistic clues as to how toxin production is controlled and potentially managed[145].

In summary, it appears that cyanobacterial-microbial interactions are generally more indicative of synergism than antagonism. Such interactions 1) occur during periods of optimal cyanobacterial growth and bloom potentials, 2) reflect mutually beneficial physiological and ecological adaptations promoting cyanobacterial dominance, and 3) appear mediated by organic molecules produced by cyanobacteria. In this regard, what we perceive to be toxins may in fact be chemical mediators of microbial interactions, bloom dynamics, and (inadvertently) water quality and health impacts. Alternatively, it is possible that toxins (e.g., microcystins) rather than being secondary metabolites, play thus far unknown roles in the regulation of cellular metabolism, with toxicity being fortuitous (or unfortunate, depending on the target organism)[146].

### Interactions with Competitors and Consumers

While cyanobacterial toxins may mediate microbial interactions, they may simultaneously inhibit potential competitors and consumers of cyanobacteria, including other phytoplankton[147,148,149,150], vascular aquatic plants[151,152], and protozoans[153,154]. However, Casanova et al.[155] concluded that normally occurring concentrations of dissolved microcystin did not affect aquatic plant germination, and that shading was the main cause of inhibition by cyanobacteria zooplankton and grazing fish.

There has been substantial study of interactions between cyanobacteria and herbivorous zooplankton, though often with conflicting results. Previous reviews of cyanobacteria-zooplankton interactions include Burns[156], Haney[157], Lampert[158], de Bernardi and Giussani[159] and Christoffersen[153]. Cyanobacterial blooms are often associated with decreases in abundance of large cladocerans of the genus *Daphnia* and increases in importance of smaller cladocerans, rotifers, and copepods[160,161,162,163,164,165,166]. There are exceptions, including the co-occurrence of large ungrazable flake-like colonies of *Aphanizomemon flos-aquae* with large *Daphnia*[167,168,169]. The more common pattern has sometimes been ascribed to increased predation on large cladocerans by planktivorous fish, which reduces grazing pressure, allowing algal blooms to occur. However, in some cases decreases in *Daphnia* appear unrelated to fish predation[170]. An alternative explanation is that the decline of large *Daphnia* is a direct effect of the cyanobacterial toxicity. There is considerable evidence that cyanobacteria can have deleterious effects on grazing zooplankton, and that large cladocerans are most adversely affected.

Inhibitory effects may be due to morphological factors (mechanical interference with feeding due to size or shape of filaments or colonies), chemical factors (toxicity, poor taste, poor nutritional value), or high densities during blooms, which may displace more nutritious algae or limit the ability of herbivores to utilize coexisting algae. Relatively few studies have attempted to distinguish among these related factors, but chemical factors appear to be more significant than mechanical interference. Fulton and Paerl[171,172,173] found that unicellular strains of *Microcystis aeruginosa* inhibited feeding rates of cladocerans on co-occurring nutritious algae more than did colonial strains, and toxic strains had the strongest inhibitory effects. The more important effect of the colonial morphology was that it made *M. aeruginosa* unavailable to cladocerans restricted to feeding on small particles (such as *Diaphanosoma brachyurum*). Rohrlack et al.[174] found evidence that the mucilage of colony-forming *M. aeruginosa* mechanically hindered *Daphnia* feeding, but lowest feeding rates occurred on a mucilage-lacking unicellular strain.

Filamentous cyanobacteria do have inhibitory effects on *Daphnia,* with the effects being strongest on larger-bodied species[169,175,176]. However, several studies indicate that chemical characteristics of cyanobacteria are more significant than the filamentous morphology. Webster and Peters[177] found both cyanobacterial filaments and cellulose fibers affected feeding behavior of zooplankton, but only filaments significantly reduced brood sizes. They concluded that mechanical effects of the cellulose fibers were not inhibitory to the cladocerans tested. Porter and Orcutt[178] found lower survivorship of *Daphnia* when fed unicells of a toxic strain of *Anabaena flos-aquae* than when fed filaments. Fulton[179] found that a toxic strain of *Anabaena flos-aquae* had stronger inhibitory effects on cladoceran feeding than did nontoxic filaments, and that most cladocerans, copepods, and rotifers tested could feed at high rates on a filamentous diatom. Other studies have also found significant consumption of some types of filamentous algae by *Daphnia* and other zooplankton[180,181]. Gilbert[182] found that extracts and mixtures of single cells and short fragments of a toxic strain of *Anabaena affinis* were just as inhibitory to growth and survivorship of cladocerans and rotifers as were intact filaments, indicating the inhibition was due to toxins, rather than to mechanical interference.

Cyanobacterial toxins can cause rapid mortality of herbivorous zooplankton[159,172,183,184,185,186,187], as well as long-term chronic effects on zooplankton growth and reproduction[148,188,189]. There is substantial variability in resistance to cyanobacterial toxins among zooplankton. Resistance to cyanobacterial toxins has been reported in rotifers[172,182,190], the small cladoceran *Bosmina longirostris*[184], and some strains of *Daphnia*[182]. Variations in susceptibility to toxins among clones of *Daphnia* indicate that toxic cyanobacteria can affect zooplankton communities at the clonal level as well as at the species level[191,192,193]. Selection acting on this clonal variability can apparently increase resistance by *Daphnia* populations to cyanobacterial toxins within decades of lake eutrophication[194]. Increased water temperature can increase the sensitivity of zooplankton to cyanobacterial toxins[193,195,196], perhaps exacerbating toxic effects at the high summer temperatures when cyanobacterial blooms often occur.

Unpalatability and poor nutritional value can also adversely affect herbivorous zooplankton[159,160]. Factors contributing to poor nutritional value include indigestibility[159,160] and deficiency of highly unsaturated fatty acids[197]. Although cyanobacteria are usually poor sole food sources for zooplankton, they can complement other food sources in mixed diets[198,199], suggesting that nontoxic cyanobacteria can contribute to zooplankton nutrition under nonbloom conditions in which they do not heavily dominate the phytoplankton community. Also, while living cyanobacteria may be poor food sources, detritus formed from decomposing cyanobacteria can be good quality food for herbivorous zooplankton[200,201].

In summary, the effects of cyanobacterial blooms on herbivorous zooplankton communities in large part reflect the interaction of chemical and mechanical inhibition. Large *Daphnia* are typically most inhibited by cyanobacteria because they are generally susceptible to toxins, are relatively unselective feeders so they cannot avoid consuming cyanobacteria, and limiting their feeding rates also limits consumption of more nutritious coexisting algae. Zooplankton species that are typically associated with cyanobacterial blooms exhibit a variety of different behaviors. One group of cyanobacteria-associated herbivores is susceptible to toxins, but avoids consumption of cyanobacteria. *Diaphanasoma bachyurum* exemplifies small cladocerans that are affected by cyanobacterial toxins, but avoid consumption of colonial or filamentous algae because they are restricted to feeding on small particles[171,172,173,179]. Copepods are also very sensitive to dissolved cyanobacterial toxins[197], but they are very selective feeders that strongly avoid consuming toxic cyanobacteria[171,172,173,179]. A second group of herbivorous zooplankton readily consume cyanobacteria, but are resistant to cyanobacterial toxins and obtain at least some degree of nutritional benefit from cyanobacteria. These include the small cladoceran *Bosmina longirostris*[184] and several rotifers[148,173,190].

### Effects on Fish and Higher Trophic Levels

Cyanobacterial toxins may affect fish and other higher trophic level consumers through three mechanisms: 1) direct exposure to cyanobacterial toxins, 2) bioaccumulation of toxins in aquatic food chains, and 3) alterations to aquatic food chain structure.

Grazing fish, such as tilapia and silver carp, are potentially significant herbivores on cyanobacteria[202]. It is therefore not surprising to find that grazing rates of herbivorous fish are inhibited by toxic *Microcystis aeruginosa*[203,204]. Predatory fish would not be expected to directly consume significant amounts of cyanobacteria, but could be affected by dissolved toxins. Fish kills associated with cyanobacterial blooms are often attributed to oxygen depletion, but cyanobacterial toxins can also kill fish[205,206], and it has been suggested that some massive fish kills during blooms of toxic cyanobacteria were caused by toxins rather than by oxygen depletion[205,206,207].

Cyanobacterial toxins can accumulate in primary consumers, including freshwater clams[208,209], crayfish[210], zooplankton, and gastropods[211]. This indicates potential transfer to higher trophic levels, but evidence is lacking. Although microcystin-LR did occur in grazing zooplankton and gastropods, Kotak et al.[211] did not find it in fish or other macroinvertebrates in the same lakes. Codd and Bell[30] radiolabeled *Daphnia magna* with microcystin-LR, then fed the cladocerans to roach and found radioactivity in tissues of the fish, but it is not certain if the radiolabel in the fish was still associated with microcystin-LR. Laurén-Määttä et al.[212] followed accumulation of microcystin in a laboratory food chain of *Microcystis aeruginosa*, *D. pulex*, and *Chaoborus* larvae, and found accumulations in the *D. pulex*, but no significant toxin accumulation in the predatory *Chaoborus*. Finally, in another laboratory study, Walsh[213] found that feeding the cyanobacterium *Anabaena flos-aquae* to the rotifer *Brachionus calyciflorus* did not affect its susceptibility to predation by the predatory rotifer *Asplanchna sylvestrii*. However, *A. sylvestrii* fed *B. calyciflorus* cultured on the cyanobacterium grew more slowly than those fed the same prey cultured on a diet of *Euglena gracilis*, suggesting potentially negative effects of this toxic cyanobacterium on trophic transfer.

Although cyanobacterial blooms produce large biomass, their toxic and other inhibitory effects on grazers could produce alterations in aquatic food web structure that would reduce productivity of higher trophic levels. A shift in zooplankton composition from large *Daphnia* to smaller or more evasive cladocerans, rotifers, and copepods may reduce the feeding success of young-of-the-year and other planktivorous fish. If cyanobacterial blooms exclude more nutritious algae, even zooplankton relatively resistant to cyanobacterial toxins may dramatically decrease in abundance, potentially leading to fish recruitment failures. Senescence or herbicidal treatment of cyanobacterial blooms may release large amounts of dissolved toxins, affecting herbivores and other organisms that would not normally consume cyanobacteria. Much of the biomass from cyanobacterial blooms probably enters microbial or detrital trophic pathways. These trophic alterations may ultimately reduce finfish and shellfish productivity.

#### Domestic Animal and Human Health Effects of Cyanotoxins

Freshwater cyanobacteria produce several toxins impacting domestic animal (pets, livestock) and human health[29,30,31,32,142,143] (Table 3). These include hepatotoxins, neurotoxins, and dermatotoxins. Hepatotoxins are the most frequently found cyanobacterial toxins in fresh and brackish waters worldwide. The most common group, the microcystins and nodularins, are cyclic peptides consisting of 7 or 5 amino acids, respectively. About 60 different structural variants of microcystins and a few nodularins are known. Potency varies from highly toxic to nontoxic depending on the specific chemical structure. Microcystins have been characterised from planktonic *Anabaena*, *Microcystis*, *Oscillatoria/Planktothrix*, *Nostoc*, *Anabaenopsis* genera, and nodularin from *Nodularia spumigena* only[31,32]. An alkaloid hepatotoxin cylindrospermopsin is produced by *Cylindrospermopsis raciborskii*, *Umezakia natans*, and *Aphanizomenon ovalisporum*[142,143].

Three families of cyanobacterial neurotoxins are known: 1) anatoxin-a and homoanatoxin-a, which mimic the effect of acetylcholine; 2) anatoxin-a(S), which is an anticholinesterase; and 3) saxitoxins, which block nerve cell sodium channels[31]. In marine waters, benthic cyanobacteria may produce toxins causing severe dermatitis among swimmers. Aplysiatoxin and debromoaplysiatoxins are protein kinase C activators and potent tumor promoters[31]. Lyngbyatoxin A exposure has caused severe oral and gastrointestinal inflammations in humans[31]. *Trichodesmium* sp. contain an uncharacterized neurotoxin (Carmichael, personal communication). Cell wall components, particularly lipopolysaccharide endotoxins (LPS), from cyanobacteria may contribute to human health problems[31,142,143].

The cyanotoxins are collectively responsible for continued widespread poisoning of wild and domestic animals plus human fatalities. While these events document the continued concern for cyanotoxins, the emerging business of fresh and marine aquaculture could be affected most. Anthropogenic inputs from agriculture, industry, and municipal wastes coupled with heavy nutrient loading by aquaculture may stimulate nuisance cyanobacterial species in aquaculture facilities. Cyanotoxins, particularly microcystins, have already had significant impacts on aqua-cultured organisms including salmon, striped bass, shrimp, and catfish (Table 3). The most well defined is the loss of net-pen reared salmon from microcystins produced by as yet unknown organisms[31]. Losses have continued since 1991 and most recently have caused salmon losses in Washington State.

### HARMFUL BLOOM MANAGEMENT

Management of nuisance blooms includes consideration of co-occurring physical, chemical, and biotic variables that synergistically and antagonistically act to control $N_2$ fixation, photosynthesis, growth, and reproductive potentials[145,214].

Widely used means of controlling blooms include (not in order of effectiveness or priority): 1) use of algicides, specifically copper sulfate; 2) nutrient input reduction and manipulation (e.g., N:P ratios); 3) vertical destratification, through mechanical mixing or bubbling; 4) enhanced water flushing to reduce retention time; and 5) biological control. Option 1 is most feasible in small impoundments, including ponds and artificial lakes used for scenic purposes (parks). This approach is neither practical nor advised in larger ecosystems, or any waters to be used for fishing, drinking, and other animal and human use. In small, relatively shallow (i.e., <5 m) systems destratification (option 3) warrants consideration. If abundant water supplies (i.e., upstream reservoirs) are available for flushing, option 4 may be possible. Biological manipulation (5) includes a number of approaches to change the aquatic food web to increase grazing pressure on cyanobacteria or to reduce recycling of nutrients. Biomanipulation approaches can include introducing fish and benthic filter feeders capable of directly consuming cyanobacteria from the water column and introduction of lytic bacteria and viruses. However, the most common biomanipulation approaches are intended to increase the abundance of herbivorous zooplankton by removing zooplanktivorous fish or introducing piscivorous fish. Alternatively, removal of benthivorous fish can reduce resuspension of nutrients from the bottom sediments. Questions have been raised about the long-term efficacy of curtailing cyanobacterial blooms by increasing grazing pressure, because this may lead to dominance by ungrazable or toxic strains[215]. Presently, biomanipulation is viewed as one component of an integrated approach to water quality management in circumstances in which nutrient reductions alone are insufficient to restore water quality[216,217,218]. Option 2 remains the most practical, economically feasible, and best long-term choice. The remainder of this section will address quantititative and qualitative considerations of nutrient manipulation strategies aimed at mitigating blooms.

### Phosphorus Management

Phosphorus inputs to aquatic ecosystems are largely attributed to three land-based sources: 1) nonpoint source surface runoff; 2) point sources, including effluents from wastewater treatment plants, industrial and municipal discharges; and 3) subsurface drainage from septic systems and groundwater. Among these, point sources have been the focus of P input cutbacks. In many watersheds, targeting point sources is justified, for they can account for a highly significant share of P loading. Point sources are frequently easiest to identify, access, regulate, and (in terms of public expenditures) finance in the context of nutrient management.

In agriculturally expanding and urbanizing watersheds, nonpoint surface and subsurface P inputs are becoming issues of increasing concern. Expanding P fertilizer use, generation, accumulation, and discharge of P-laden animal waste, soil disturbance, and loss due to conversion of forests and grasslands to rowcrop and other intensive farming operations, and the proliferation of septic systems accompanying human population growth are rapidly increasing the importance and burden of nonpoint P loading. In many agricultural and urban watersheds, nonpoint sources can account for at least 50% of annual P loading. Because of their diffuse nature, they are far more difficult to identify and address from a nutrient management perspective. Nonpoint source P management will be an increasingly important nutrient control strategy in these watersheds.

As with nitrogen (see below), the manner in which P is discharged to P-sensitive waters is of paramount concern. Key considerations include: 1) total annual (i.e., chronic) P loading, 2) shorter-term seasonal and event-based pulse (i.e., acute) P loadings, 3) particulate vs. dissolved P loading, and 4) inorganic vs. organic P loading. In terms of overall ecosystem P budgets and long-term responses to P loadings (and reductions), annual P inputs are of fundamental importance. In terms of specific biological community responses (i.e., primary productivity and bloom formation), seasonal and shorter-term acute loading events are of critical, and at times, overriding importance. Experience has taught us that when and where P inputs occur can determine the difference between a bloom-plagued vs. bloom-free conditions. For example, if a large spring P discharge event precedes a dry, stagnant summer in a relatively long residence time water body, the spring P load will be available to support summer bloom development and persistence. Effective exchange and cycling between the water column and bottom sediments can retard P transport and hence retain P as it travels through a water body. As a result, acute P inputs during relatively high flow periods may be retained longer in the system than would be estimated based on water flushing/retention times. In effect, the water body can exhibit both rapid biological responses to, and a distinct "memory" for, acute P loads.

Unlike N, P exists in relatively few dissolved and particulate forms in natural waters. Furthermore, there are no gaseous forms of P that can be gained or lost by the system via biological transformations. Therefore, we need to be mainly concerned about dissolved and particulate forms of inorganic and organic P. Dissolved inorganic P (DIP) exists exclusively as orthophosphate ($PO_4^{3-}$). This form can be rapidly assimilated by phytoplankton, and in the case of most bloom-forming cyanobacteria, is accumulated intracellularly as polyphosphates. Polyphosphates can serve as internal stores of P, for subsequent use in the event of ambient P depletion[219]. Dissolved organic P (DOP) can be a significant fraction of the total dissolved P pool. DOP can be assimilated by a variety of microorganisms, including microalgae and cyanobacteria, although not as rapidly as DIP[220]. A large fraction of the assimilated DOP is microbially recycled to DIP, making it available for subsequent utilization.

The role of particulate P (inorganic or organic) in aquatic production and nutrient cycling dynamics is less well understood. Particulate P (PP) may provide a source of DIP and DOP via desorption and leaching processes, and it may serve as a sorption and precipitation site for DIP. PP therefore exists in dynamic equilibrium with the dissolved phases of P. It can be concluded that PP can serve as a source of biologically available P and as such play an important role in P cycling and productivity. Furthermore, on the ecosystem-level, sedimented PP serves as an important source of stored P for subsequent release, especially during hypoxic and anoxic periods, when large amounts of DIP are released from the sediments. These sources of stored and recycled P can serve to support additional phytoplankton blooms. Clearly, *both* dissolved and particulate forms of P input must be accounted for when formulating and managing P inputs and N:P ratios.

### Nitrogen Management

N exists as dissolved and particulate, aqueous and gaseous forms. The multiple chemical forms of N create both scientific and management challenges because virtually all the forms are biologically available and readily exchanged within and between the water column and sediment. In addition, biological $N_2$ fixation and denitrification control the exchange (inputs and outputs, respectively) between inert gaseous atmospheric dinitrogen and biologically available combined N forms.

Combined forms of N include dissolved inorganic N (DIN; ammonium [$NH_4^+$], nitrate [$NO_3^-$], and nitrite [$NO_2^-$]), dissolved organic N (DON; e.g., amino acids and peptides, urea, organonitrates), and particulate organic N (PON; polypeptides, proteins, complex organic matter, organic detritus). These sources are supplied as diffuse nonpoint and distinct point sources. Nonpoint sources include surface runoff, atmospheric deposition and groundwater, while point sources are dominated by municipal, agricultural, and industrial wastewater effluent. In most rural and agricultural settings, nonpoint sources of N input tend to dominate (>50% of total N loading), while in urban centers, point sources often dominate. Agricultural and urban runoff, atmospheric deposition, and municipal/industrial effluents contain a variety of organic and inorganic chemical species in dissolved and particulate forms. These sources constitute a dynamic mixture of biologically available DIN, DON, and PON that plays a critical role in eutrophication. Depending on sources, chemical makeup, delivery mechanisms, and spatial distribution of N inputs, ecosystem response can vary dramatically.

N inputs are highly dynamic, reflecting land use, population and economic growth, and changes therein. For this reason, N has often been referred to as the "currency" of eutrophication in N-sensitive estuarine and coastal waters. The means and routes by which human N sources impact and mediate eutrophication are changing. Among the most rapidly growing (both in amount and geographic scale) sources of anthropogenic N loading are surface runoff, groundwater, and atmospheric deposition. Atmospheric N loading is a frequently overlooked, but expanding source of *new* N loading to N-sensitive waters. In coastal North Carolina, for example, the combined emissions of fossil fuel combustion ($NO_x$) and volatilization of $NH_3$ from animal waste (wastewater lagoons and land-applied) are a major (>30% of *new* N loading), rapidly growing source of biologically available N[61]. Surface and groundwater N releases from expanding animal operations and urbanization are of additional concern. The diversity and highly dynamic nature of these N sources provide several chemically distinct nitrogenous compounds such as nitrite, nitrate, ammonium, urea, and other organic N compounds.

The contribution of groundwater and atmospheric N to N-sensitive estuarine and coastal watersheds will increase substantially in the next millennium, when nearly 70% of North American and European populations will reside within 50 km of the coast. A significant fraction of atmospheric N is directly deposited to estuarine and coastal waters, bypassing the estuarine N "filter". In many locations, including the Eastern Seaboard of the U.S., atmospheric N is among the dominant sources of anthropogenic N to the coastal zone. When and where anthropogenic N inputs are intercepted are critical factors in determining ecosystem sensitivity, water quality responses and resourcefulness in response to upstream (watershed) and upwind (airshed) N enrichment. The biogeochemical, ecological, and cultural ramifications of this previously "out of sight out of mind", growing new N source must be clarified and incorporated in our understanding of estuarine and coastal ecosystem function and management.

Chronic anthropogenic N and P loading of our rivers, lakes, and estuaries has caused state and federal agencies to assign nutrient-sensitive status and impose total mean daily loads (TMDLs) to these waters. These designations require formulation and implementation of nutrient input reduction strategies aimed at arresting eutrophication and lowering nuisance bloom potential. The Neuse River Estuary, North Carolina has been the site of periodic massive blooms of the non-$N_2$-fixing, toxic, surface scum-former *Microcystis aeruginosa*, with incursions of the filamentous non-$N_2$-fixer *Oscillatoria* spp. Dominance by these non-$N_2$-fixers attests to the current N "overload situation"[12,221]. Dilution bioassays indicated that watershed loading of N would need to be reduced by 30 to 40% to obtain N-limited conditions during the critical spring bloom initiation period. A complicating aspect from nutrient dynamics and management perspectives is the presence (as subdominants) of $N_2$-fixing cyanobacteria, *Anabaena* and *Aphanizomenon*. This fact, combined with evidence that P loading also is excessive[12,222,223], suggests that if N loading is reduced by 30 to 40% without parallel P reductions, there may be potential for replacing non-$N_2$-fixing *Microcystis* with $N_2$-fixing *Anabaena* or *Aphanizomenon* blooms. Results from bioassays in which N was reduced but P was not confers the potential for selective stimulation of $N_2$-fixing cyanobacteria in freshwater and oligohaline segments of the eutrophying Neuse River and St. Johns River (Florida) systems (Paerl et al., in preparation). Work is currently under way to formulate parallel N and P reductions aimed at avoiding replacement of one type of cyanobacterial nuisance with another.

### CONCLUDING REMARKS

Cyanobacterial water blooms are a worldwide phenomenon, and as such are regulated by an interplay of geographically and ecologically diverse environmental variables. The long evolutionary history of cyanobacterial bloom species has dictated a high degree of tolerance and adaptability to both short-term (i.e., diel, seasonal, decadal) and long-term (geological) environmental change, essentially making these photosynthetic prokaryotes a "group for all seasons".

In this review, we have explored the myriad physical, chemical, and biotic factors implicated in the development, proliferation, and expansion of cyanobacterial blooms. Despite their seemingly infinite adaptation to environmental change on both geological and biological time scales, cyanobacterial nuisance characteristics (e.g., large anoxia-generating and toxic blooms) are, to a large extent, products of human alteration of water- and airsheds for aquatic environments. The most notable and controllable alterations include: 1) nutrient (especially N and P) enrichment; 2) hydrological changes, including freshwater diversions, the construction of impoundments such as reservoirs, and excessive water use for irrigation, drinking supplies, flood control, etc., all of which affect water residence time or flushing rates; 3) biological alterations of aquatic ecosystems, including manipulations of grazers (from zooplankton to fish); and lastly 4) the use and introduction of toxins and xenobiotic compounds (e.g., heavy metals, herbicides and pesticides, industrial and domestic chemicals, antibiotics, and other synthetic growth regulators), all of which exert phytoplankton community growth and composition altering effects.

Effective long-term management of nuisance, particularly toxin-producing, cyanobacterial blooms must address the above-mentioned suites of environmental factors, in combination with knowledge of the ecological and physiological adaptations that some species possess to circumvent certain controls derived from our knowledge of these factors. Examples include: 1) the ability of certain $N_2$ fixers to take advantage of N-limited conditions; 2) the ability of buoyant taxa to counteract mixing and other means of man-induced destratification aimed at minimizing cyanobacterial dominance; and 3) specific mutualistic and symbiotic associations that cyanobacteria have with other microorganisms, higher plants and animals, which may provide clues as to the roles toxins and other chemical factors play in shaping biotic community structure and function.

Progress in identifying and understanding the roles toxins and other metabolites play in the physiology and ecology of bloom-forming cyanobacteria will be achieved by integrating physiological, toxicological, and ecological perspectives and expertise. This includes hypothesis testing and problem solving using interdisciplinary experimental, monitoring, and assessment approaches. In addition, the synthesis of well-defined laboratory experimental work with ecosystem-level studies utilizing similar techniques and measurements will be essential to unraveling the complexity of environmental regulation of cyanobacterial bloom dynamics. We are at the threshold of more holistic approaches to environmental problem solving. In this regard, the incorporation of novel analytical and molecular quantification and characterization techniques in environmental biology and management will prove invaluable.

### ACKNOWLEDGEMENTS

Research and logistic support were provided by the National Science Foundation (Projects OCE 94-15985, DEB 9815495), the U.S. Department of Agriculture (Project NCR-9600509), the U.S. Environmental Protection Agency (Project R825243-01-0), the National Oceanographic and Atmospheric Administration (NOAA/North Carolina Sea Grant Program; RMER 30), and the St. Johns Water Management District, Florida. We appreciate the technical assistance from M. Go, B. Peierls, D. Whitall, and R. Barnes.

| | | |
|---|---|---|
| Received: | January | 22, 2001 |
| Revised: | February | 7, 2001 |
| Accepted: | February | 8, 2001 |
| Published: | April | 4, 2001 |
